# White Matter Networks of Phonological Awareness in Chinese Readers

**DOI:** 10.1002/brb3.70781

**Published:** 2025-09-21

**Authors:** Xinyue Zhang, Yueye Zhao, Siyu Chen, Zi‐Gang Huang, Jingjing Zhao

**Affiliations:** ^1^ School of Psychology Shaanxi Normal University Shaanxi China; ^2^ The Key Laboratory of Biomedical Information Engineering of the Ministry of Education, Institute of Health and Rehabilitation Science, School of Life Science and Technology Xi'an Jiaotong University Xi'an China; ^3^ Research Center for Brain‐Inspired Intelligence Xi'an Jiaotong University Xi'an China; ^4^ Department of Psychology The Chinese University of Hong Kong Hong Kong China; ^5^ Brain and Mind Institute The Chinese University of Hong Kong Hong Kong China

**Keywords:** character reading, fusiform gyrus, middle temporal gyrus, phonological skills, white matter network

## Abstract

**Introduction:**

In our previous study, we have identified white matter subnetworks linked to phonological processing deficits (e.g., subnetworks centered at the left middle temporal gyrus) in dyslexic children from alphabetic languages using a data‐driven hub‐based white matter network analysis approach. Yet, white matter subnetworks associated with phonological processing skills in individuals from nonalphabetic languages (e.g., Chinese) have never been studied. This study aims to identify hub‐related white matter networks associated with phonological processing skills in Chinese readers.

**Methods:**

Sixty‐five Chinese‐speaking adults were classified into good readers (*n* = 37) and poor readers (*n* = 28) based on the severity of self‐reported reading difficulties and dyslexia symptoms, as assessed by the Chinese adaptation of the Adult Reading History Questionnaire (C‐ARHQ). We explored hub‐related networks corresponding to phonological processing skills among the participants. Mediation analysis was further conducted to examine the relationship between white matter networks, phonological awareness, and character reading ability of these adults.

**Results:**

We observed structural connectivity of two hub‐related white matter networks accounted for individual differences of phonological awareness in Chinese readers: white matter networks surrounding the left middle temporal gyrus and the left fusiform gyrus. Follow‐up mediation analysis revealed that the two white matter networks further contributed to the character reading ability of Chinese readers through phonological awareness.

**Conclusions:**

The current study provides the first‐hand empirical evidence for the white matter network of phonological processing skills in Chinese readers. The findings offer an important cross‐linguistic insight into the white matter network corresponding to phonological processing in nonalphabetic languages.

## Graphical abstract

White matter networks surrounding the left middle temporal gyrus and the left fusiform gyrus account for individual differences in phonological awareness, which completely mediates the relationship between these networks and Chinese character reading. This study provides first‐hand empirical evidence for the white matter networks of phonological awareness in Chinese readers.

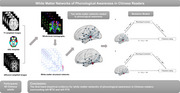

## 1 Introduction

Reading is a multifaceted skill that includes several cognitive processes (Snowling and Melby 2016). Individuals facing reading challenges typically exhibit impairments in phonological deficits (Ramus and Szenkovits 2008; Ramus et al. 2018). Such individuals exhibit poor performance in tasks assessing phonological awareness (PA) and rapid automatized naming (RAN). Yet, neural underpinnings of phonological processing skills have not been fully understood.

Phonological awareness, a critical aspect of phonological processing, entails the deliberate segmentation and manipulation of sounds made in speech, along with mapping these sounds onto their corresponding written symbols (Bradley and Bryant 1985; Castles and Coltheart 2004; Hulme et al. 2005). Research across diverse writing systems, both alphabetic and nonalphabetic languages, has consistently highlighted PA as a vital contributor to reading ability development (e.g., Ziegler and Goswami 2005; Shu et al. 2008). PA deficiency is frequently observed as a core characteristic of developmental dyslexia in various linguistic contexts, including both European languages and Chinese (Landerl et al. 2013; Ding et al. 2024, 2025). Additionally, a number of studies have found a significant connection between PA and the ability to read Chinese characters (Shu et al. 2008; Tong et al. 2011; Newman et al. 2011; Pan et al. 2016; Cao et al. 2020). RAN pertains to the capacity for rapidly naming a sequence of familiar items—including numbers, letters, characters, images, or colors—which is an indicator of phonological processing speed (Denckla and Rudel 1976; Norton and Wolf 2012; Wolf and Bowers 1999; Saksida et al. 2016). RAN has been found to contribute to reading skills related to word reading (Wolf et al., 2002) and is known as another well‐established predictor of dyslexia in both alphabetic and nonalphabetic languages (Landerl et al. 2013; Ding et al. 2024, 2025).

In the last 20 years, diffusion tensor imaging (DTI) has been extensively utilized to clarify the neural mechanisms associated with phonological processing abilities (Klingberg et al. 2000; Deutsch et al. 2005; Rimrodt et al. 2010; Langer et al. 2017; Vandermosten et al. 2015). Notably, studies have identified the arcuate fasciculus (AF) as being significantly associated with phonological skills (Keller and Just 2009; Saygin et al. 2013; Martins et al. 2024). Research on preschool and primary school children showed a positive association between AF integrity and PA (Yeatman et al. 2011; Vandermosten et al. 2012; Vanderauwera et al. 2015; Reynolds et al. 2019; de Vos, 2020). Su et al. (2018) also found that the AF was correlated with PA in Chinese children. Yet, most of these neuroimaging investigations of white matter structural connectivity have predominantly employed voxel‐level methodologies or tractography methods to examine discrete fiber pathways. Emerging evidence suggests that a particular cognitive function is not involved in just a single white matter tract but is supported by a comprehensive neural network of multiple regions.

Graph theory presents a simple yet powerful method of connectome to explore the neural underpinnings of phonological deficits from the brain network perspective (Bullmore and Bassett 2011; Sotiropoulos and Zalesky 2019). In contrast to tract‐specific imaging techniques, the construction of macroscale connectomes reveals the global brain networks’ organizational features, including degree, small‐worldness, efficiency, and modularity. These features are applicable to the description and comparison among different brain structural and functional networks (Bassett and Bullmore 2009; Bullmore and Bassmore 2011; Van Den Heuvel and Sporns 2013). Complementary to traditional tractography methods, graph theory has been used to explore white matter networks related to reading ability and phonological skills from a whole‐brain perspective. For instance, using a network‐based analysis, our research team found that French dyslexic children presented lower connections than control children among the occipital‐temporal‐parietal subnetwork, which mainly involves a dorsal pathway of AF and a ventral pathway linked to the inferior longitudinal fasciculus (ILF) (Lou et al. 2019). Applying a hub‐based network analysis method, our research team further revealed that the networks around the left middle temporal gyrus (MTG) explained individual differences in phonological processing accuracy in dyslexic children (Liu et al. 2022) and the white matter network surrounding the right fusiform gyrus (FFG) accounted for the severity of phonological decoding ability in dyslexic children (Liu et al. 2021).

As a nonalphabetic language, Chinese reading differs fundamentally from alphabetic languages (e.g., English, French). Its unique linguistic properties (e.g., morpheme‐syllable mapping, visual complexity) may shape distinct neural mechanisms, making it an ideal model for studying cross‐language reading networks. While research on alphabetic writing systems has shown the key role of the left temporal‐parietal network in phonological processing (Liu et al. 2022; Turker et al. 2023; Vandermosten et al. 2012), the neural networks associated with phonological processing in Chinese readers were still unknown. Although previous studies have reported that the left MTG was important for representing Chinese phonological information (Li et al. 2022), no research has yet systematically explored whether the white matter network around MTG was also responsible for Chinese PA.

In sum, prior research has found the networks centered at the left MTG being implicated in phonological processing in alphabetic readers. Nevertheless, research on the white matter network associated with the phonological processing skills of Chinese‐speaking individuals was rare. Our study intended to explore the connectivity of hub‐based white matter subnetworks linked to phonological skills in Chinese young adults.

## 2 Materials and Methods

### 2.1 Participants

This study recruited 65 Shaanxi Normal University undergraduates (aged 17–19 years old). All individuals were right‐handed native Chinese speakers. They had typical hearing and vision abilities. None had any history of neurological or psychological disorders. The threshold used in the Chinese Adult Reading History Questionnaire (C‐ARHQ) to indicate a history of reading difficulties was set at 0.36 (He et al. 2025). According to this, 28 participants in the current study were defined as poor readers (score above 0.36), while 37 were good readers. Both groups were matched for age, sex, and nonverbal IQ. Table 1 details demographics. The Shaanxi Normal University Ethics Committee approved the study (HRHR2024‐04‐01). All subjects provided written informed consent prior to magnetic resonance imaging (MRI) and behavioral investigation.

**TABLE 1:** Demographic data and behavioral results of good readers and poor readers.

	Good readers (N=37)	Poor readers (N=28)		
	Mean (*SD*)	Mean (*SD*)	Test statistics	
***Subject characteristics***				
Sex (male/female)	14/23	12/16	*χ*(1)^2^ = 0.167	*p* = 0.683
Age(years)	18.08 (0.28)	18.04 (0.58)	*t*(63) = 0.384	*p* = 0.703
Non‐verbal IQ	56.54 (2.35)	57.21 (2.41)	*t*(63) = −1.132	*p* = 0.262
***Reading ability***				
Character Reading	89.35 (10.70)	75.86 (15.73)	*t*(63) = 3.906	*p* < **0.001** ^***^
***Phonological awareness***				
Phoneme deletion	14.62 (1.67)	13.68 (1.96)	*t*(63) = 2.088	*p* = **0.041** ^*^
Spoonerism I	13.46 (2.63)	11.75 (3.35)	*t*(63) = 2.305	*p* = **0.024** ^*^
Spoonerism II	15.05 (1.27)	14.11 (1.77)	*t*(63) = 2.513	*p* = **0.015** ^*^
***Rapid automatized naming (RAN)***				
RAN digits	9.45 (1.67)	9.82 (1.77)	*t*(63) = −0.849	*p* **=** 0.399
RAN objects	20.94 (2.52)	21.91 (2.94)	*t*(63) = −1.434	*p* **=** 0.156
RAN colors	23.37 (2.84)	23.64 (3.55)	*t*(63) = −0.338	*p* **=** 0.736

Bold values indicate significant group differences (*p* < 0.05, two‐tailed).

* *p* < 0.05

** *p* < 0.01

*** *p* < 0.001.

### 2.2 Behavioral Measures

All participants took behavioral tests to assess their character reading and phonological processing skills. The Raven IQ test measured nonverbal intelligence. The character reading test included 150 Chinese characters (Zhao et al. 2025). Three tasks, phoneme deletion, spoonerism I, and spoonerism II, assessed PA. In the phoneme deletion task, participants had to speak out what was left after removing a phoneme of a syllable. Two items removed the first consonant (e.g., /an4/ from /lan4/). Four items removed the middle vowel (e.g., /si4/ from /sai4/). Ten items removed the final sound (e.g., /gan1/ from /gang1/). Each right answer gave one point, and the highest score was 16. A previous study found this test had a Cronbach's α of 0.90 (Cheng et al. 2021). In Spoonerism I task, two Chinese syllables were spoken to participants, and participants formed a new syllable by using the first syllable's initial consonant and the second syllable's vowel. The task had 16 items, with high reliability (Cronbach's α = 0.9) (Cheng et al. 2021). The more difficult Spoonerism II task involved swapping the beginning consonants of two syllables, such as /bai2/ and /mao1/ into /bao1/ and /mai2/ (Cronbach's α = 0.78; He et al. 2025). RAN tests measured phonological processing speed. Three categories—digits, colors, and pictures—were assessed in the RAN tests (Ding et al., 2024, 2025). Every subtest had 40 items in eight rows and five columns. Participants were instructed to name these items when they were displayed in a pseudo‐random sequence as quickly and precisely as they could. The tests were conducted twice, with the mean of completion durations from both tests as the final RAN scores. These tests had high test–retest reliability, with coefficients of 0.83 (digits), 0.70 (colors), and 0.82 (pictures), respectively (He et al. 2025).

PA was calculated by averaging phoneme deletion, Spoonerism I, and Spoonerism II *Z*‐scores. RAN was calculated through the averaging of digit, picture, and color *Z*‐scores. Directions were adjusted with positive *Z*‐scores signifying performance that exceeded the average.

### 2.3 Image Acquisition

In the Brain Imaging Centre at Xi'an Jiaotong University, we used a German Siemens Trio 3‐Tesla MRI scanner to collect neuroimaging data. A manufacturer‐provided foam pad reduced head motion, and earplugs reduced scanner noise. Participants were supine in the scanner's whole‐body gradient field (40 mT/m, 200 T/m/s) with a 32‐channel head coil for best signal reception. T1‐weighted images and diffusion‐weighted images were collected. Using a magnetization‐prepared rapid acquisition gradient echo (MPRAGE) sequence, T1‐weighted images were obtained with the following parameters: acquisition matrix = 230 × 230 × 192, flip angle = 9°, repetition time (TR) = 2300 ms, echo time (TE) = 2.61 ms, and voxel dimensions = 0.9 × 0.9 × 0.9 mm^3^.

A multi‐shell diffusion‐weighted imaging dataset was acquired using a single‐shot echo planar imaging sequence with two nonzero *b*‐values (1000 and 2000 s/mm^2^). For each *b*‐value, 64 diffusion‐weighted volumes were collected using unique, noncollinear gradient directions. All diffusion‐weighted volumes were obtained with phase encoding in the anterior‐to‐posterior (AP) direction. In addition, six b0 images (*b* = 0 s/mm^2^, i.e., no diffusion weighting) were collected. Three of these were obtained with AP phase encoding, and the remaining three with reversed phase encoding (posterior‐to‐anterior). Seventy slices per volume were acquired, with each slice 1.7 mm thick. The field of view was 218 × 218 mm^2^ with a 128 × 128 acquisition matrix, and 1.7 × 1.7 × 1.7 mm^3^ voxels. TR was 12,200 ms, and TE was 129 ms.

### 2.4 Diffusion Tensor Imaging Analysis

MRI data were processed using PANDA (Cui et al. 2013), a toolbox built on FSL (version 6.0.1). The initial step involved converting the raw DICOM files into a 4D NIfTI format using MRIcron's dcm2nii conversion. Next, a brain extraction procedure was applied to strip away non‐brain tissue and generate a brain mask for each subject. Eddy current correction was then performed to address image distortions and head motion artifacts. Each diffusion‐weighted image was registered to the b0 reference image, and the diffusion gradient directions were realigned accordingly. Fitting the diffusion tensor to head motion corrected data yielded fractional anisotropy (FA), which measures white matter coherence and integrity (Alexander et al. 2007; Beaulieu. 2002; Jones et al., 2013). The standard FMRIB58_FA template normalized each subject's FA maps to Montreal Neurological Institute (MNI) space. All raw diffusion‐weighted (DW) images and registration underwent a manual inspection to exclude participants with significant artifacts (e.g., motion artifacts) or incomplete data acquisition in order to ensure good quality (Liu et al. 2022). Deterministic fiber tracking method employed by Continuous Tracking (FACT) method (http://trackvis.org/dtk) was used to track whole‐brain fibers with an angular threshold of 45° and an FA threshold between 0.2 and 1.

### 2.5 Network Node Definition

The Automated Anatomical Labelling (AAL) atlas defined white matter network nodes (Tzourio‐Mazoyer et al. 2002). Each subject's cortical gray matter, excluding the cerebellum, was divided into 90 regions of interest (ROIs). To remove ocular and neck artifacts from T1 images, PANDA's Brain Extraction tool was used. Subsequently, further spatial normalization was carried out using the structural T1‐weighted images. Each subject's T1‐weighted anatomical scan was linearly coregistered to its corresponding FA map in the native diffusion space. The T1 images, after coregistration, were subjected to a nonlinear transformation to match the standard ICBM‐152 brain template (Montreal Neurological Institute space) for spatial normalization. Using the inverse of this transformation, the AAL atlas defined in MNI space was mapped into each individual's native DTI space.

### 2.6 Identification of the Backbone Network in Controls

Following Gong et al. (2009), we defined the backbone network based on the good readers to ensure stable and highly consistent cortical connections. This approach has been validated in previous studies as stable and unbiased, which ensured that the network structure was fixed (Liu et al. 2021, 2022). For each potential pair of cortical nodes, we applied a one‐tailed nonparametric sign test under the null hypothesis of no connection between them (i.e., fiber bundle count = zero). The Bonferroni correction was applied for multiple comparisons (C_90_^2^ = 4005 pairs of regions, *p* < 0.05/4005 ≈ 1.25×10^−5^) to create a symmetric binarized matrix that preserved 1816 tracts. The finished network has 22.42% sparsity, which is within a range shown to support stable graph metrics in structural brain networks (Dennis et al. 2012).

### 2.7 Statistical Analysis

The mean FA of the backbone network was extracted for both the poor readers and the good readers groups using the backbone network mask derived from the good reader group. Additionally, the nodal degree values for each region of the AAL atlas were calculated by summing the FA values of all edges connected to each respective node. Character reading, PA, and RAN scores were compared using t‐tests. An analysis of covariance (ANCOVA) was performed on nodal degree values across all 90 AAL regions to find structural white matter variations between groups. To discover brain hubs connected to reading abilities, partial correlations between nodal degree values and character reading, PA, and RAN were examined across the whole group and within each group after controlling for sex, age, and nonverbal IQ. After that, a Fisher *Z* test was conducted on the correlation coefficients in the two groups to explore whether there was a significant difference. To control for multiple comparisons, the Benjamini–Hochberg (BH) false discovery rate (FDR) procedure was applied (Benjamini and Hochberg 1995). The significance threshold was set at *q* < 0.05. A hierarchical linear regression analysis was conducted to examine the independent contributions of specific brain regions in predicting phonological abilities. The mediation model between brain measurements, PA, and character reading was examined using SPSS 27.0 PROCESS 4.1 mediation analysis. Nodal degree values were the independent variable, PA was the mediator, and character reading was the dependent variable. Age, sex, and IQ were included as covariates in all mediation models. Bootstrapping with bias‐corrected 95% confidence intervals (CI) was employed to assess the significance of mediation effects, utilizing 1000 bootstrap samples (McCartney et al. 2006). This approach offers the most accurate estimation of CI and maximizes statistical power for detecting mediation effects (MacKinnon et al. 2004).

## 3 Results

### 3.1 Demographics and Behavioral Results

Table 1 shows demographic and behavioral statistics of the two groups. Poor and good readers showed no significant difference in gender, age, and nonverbal IQ. Character reading and PA were worse in poor readers than in good readers.

### 3.2 Group Differences in White Matter

After controlling for sex, age, and nonverbal IQ, FA nodal degree values of the left middle frontal gyrus (MFG.L), right Rolandic operculum (ROL.R), left superior frontal gyrus medial orbital (ORBsupmed.L), left gyrus rectus (REC.L), right cuneus (CUN.R), left caudate nucleus (CAU.L), right putamen (PUT.R), right pallidum (PAL.L), and left middle temporal gyrus (MTG.L) were lower in poor readers compared with good readers (see Table S1). However, none of the comparisons across groups survived BH FDR correction (q < 0.05).

### 3.3 Partial Correlation Between Nodal Degree Values and Reading Abilities

Since there was no group difference between the two groups, partial correlations were computed for all participants between character reading, PA, and RAN and nodal degree values of the 90 AAL regions (see in Table S2). Nodal degree values of two AAL regions were significantly linked with PA after BH FDR correction (controlling for sex, age, and nonverbal IQ): left MTG (*r* = 0.56, *p* = 0.000003, FDR‐adjusted *p* = 0.00027) and left FFG (*r* = 0.449, *p* = 0.0003, FDR‐adjusted *p* = 0.0129). None of these regions’ nodal degree was linked with character reading and RAN (see Table S2). Additionally, Fisher's Z test revealed the correlation coefficient between the left FFG and PA and the correlation coefficient between the left MTG and PA in the two groups had no difference (left FFG: *p* = 0.999; left MTG: *p* = 0.912).

**FIGURE 1:**
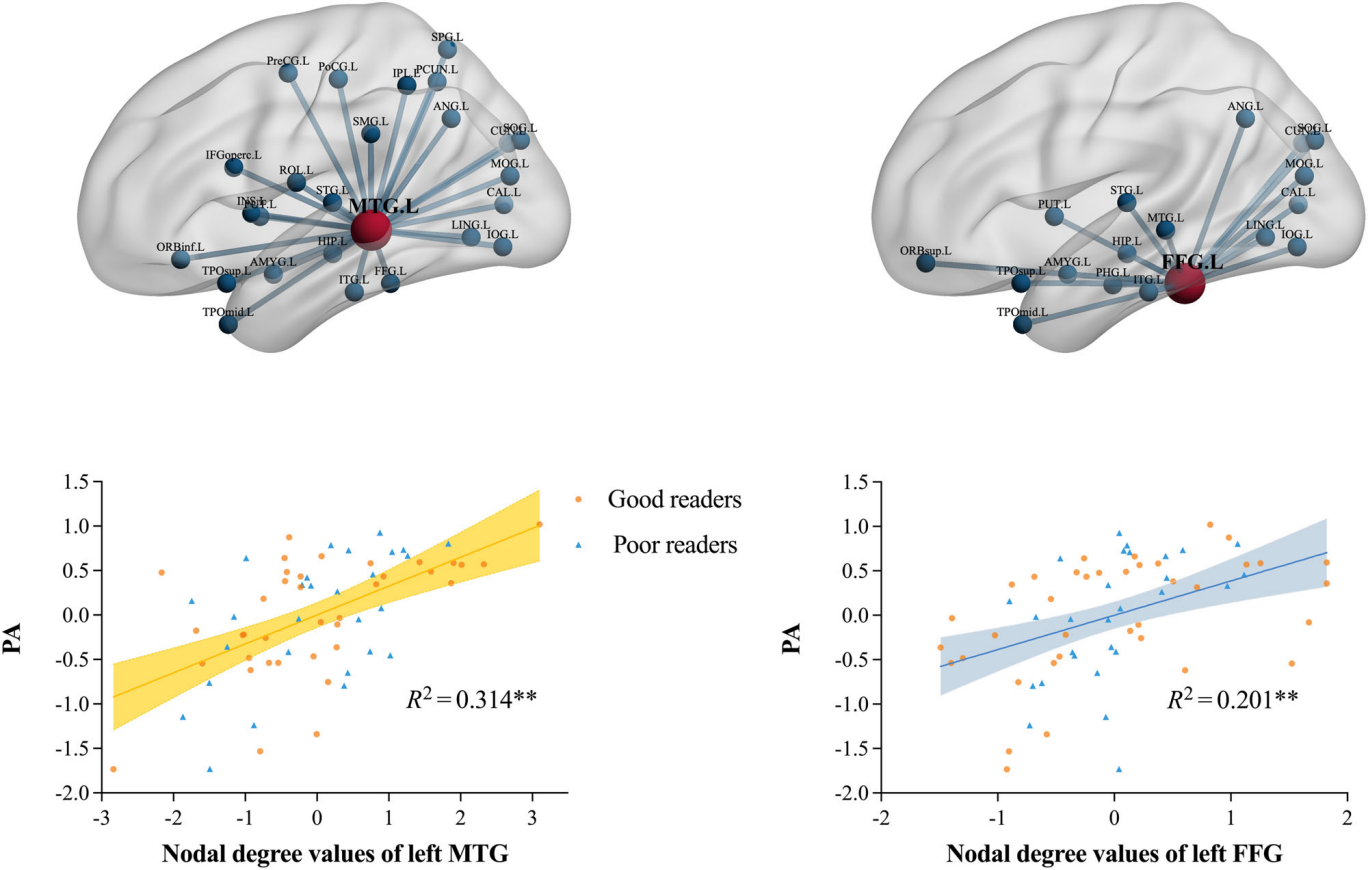
Left‐top: The hub and white matter subnetwork corresponding to phonological awareness (PA)—left middle temporal gyrus (MTG), connected with left precentral gyrus (PreCG), left inferior frontal gyrus opercular part (IFGoperc), left inferior frontal gyrus orbital part (ORBinf), left Rolandic operculum (ROL), left insula (INS), left hippocampus (HIP), left Amygdala (AMYG), left calcarine fissure and surrounding cortex (CAL), left cuneus (CUN), left lingual gyrus (LING), left superior occipital gyrus (SOG), left middle occipital gyrus (MOG), left inferior occipital gyrus (IOG), left fusiform gyrus (FFG), left postcentral gyrus (PoCG), left superior parietal gyrus (SPG), left inferior parietal, but supramarginal and angular gyri (IPL), left supramarginal gyrus (SMG), left angular gyrus (ANG), left precuneus (PCUN), left lenticular nucleus putamen (PUT), left superior temporal gyrus (STG), left temporal pole superior temporal gyrus (TPOsup), left MTG, left temporal pole middle temporal gyrus (TPOmid) and left inferior temporal gyrus (ITG). Right‐top: The hub and white matter subnetwork corresponding to phonological awareness (PA)—left fusiform gyrus (FFG), connected with left superior frontal gyrus orbital part (ORBsup), left HIP, left parahippocampal gyrus (PHG), left AMYG, left CAL, left CUN, left LING, left SOG, left MOG, left IOG, left ANG, left PUT, left STG, left TPOsup, left MTG, left TPOmid, left ITG. Left‐bottom: Scatterplot of the correlation between residuals of the nodal degree values of the left MTG and residuals of the PA scores in whole groups (controlling for sex, age, and Raven IQ). Right‐bottom: Scatterplot of the correlation between residuals of the nodal degree values of the left FFG and residuals of the PA scores in whole groups (controlling for sex, age, and Raven IQ). The coefficients of *R* ^2^ are statistically significant. ^**^ *p* < 0.01.

White matter subnetworks connected with the left MTG and the left FFG are plotted in Figure 1, respectively. The left MTG is connected to 25 regions, including the left precentral gyrus (PreCG), left inferior frontal gyrus opercular part (IFGoperc), left inferior frontal gyrus orbital part (ORBinf), left Rolandic operculum (ROL), left insula (INS), left hippocampus (HIP), left Amygdala (AMYG), left calcarine fissure and surrounding cortex (CAL), left cuneus (CUN), left lingual gyrus (LING), left superior occipital gyrus (SOG), left middle occipital gyrus (MOG), left inferior occipital gyrus (IOG), left FFG, left postcentral gyrus (PoCG), left superior parietal gyrus (SPG), left inferior parietal (IPL), left supramarginal gyrus (SMG), left angular gyrus (ANG), left PCUN, left lenticular nucleus putamen (PUT), left superior temporal gyrus (STG), left temporal pole superior temporal gyrus (TPOsup), left MTG, left temporal pole MTG (TPOmid) and left inferior temporal gyrus (ITG) as shown in Figure 1 (left‐top). The left FFG is connected to 17 regions, including the left superior frontal gyrus orbital part (ORBsup), left HIP, left Parahippocampal gyrus (PHG), left AMYG, left CAL, left CUN, left LING, left SOG, left MOG, left IOG, left ANG, left PUT, left STG, left TPOsup, left MTG, left TPOmid, left ITG, as shown in Figure 1 (right‐top). Figure 1 (left‐bottom) shows the scatterplot of the correlation between the left MTG node degree values and PA across all the participants. Figure 1 (right‐bottom) shows the scatter plot of the correlation between the left FFG node degree values and PA across all the participants.

### 3.4 Regression Analysis

We conducted a hierarchical linear regression analysis to further evaluate whether the nodal degrees of the left MTG and FFG were independently associated with PA. PA was entered into the model as a dependent variable. Control factors included gender, age, and nonverbal IQ. The left FFG and left MTG nodal degree values were entered into the model concurrently as independent variables in the second stage. This statistical analysis was based on existing research (Liu et al. 2021, 2022); it could be used to test whether specific brain regions (left MTG/FFG) had unique contributions to PA. To examine potential multicollinearity among the independent variables, collinearity diagnostics were performed. All variance inflation factor (VIF) values ranged from 1.036 to 1.321, and all tolerance values exceeded 0.7, indicating no significant multicollinearity concerns. In Table 2, after controlling for sex, age, and nonverbal IQ (Δ*R^2^* = 0.100), significant predictions were observed from both the left FFG and the left MTG nodal degrees to PA (Δ*R^2^* = 0.190; left MTG: *β* = 0.512, *p* < 0.001; left FFG: *β* = 0.216, *p* = 0.044). It should be noted that sex also predicted PA (*β* = 0.300, *p* = 0.004).

**TABLE 2:** Hierarchical regression models of phonological awareness predicted by the nodal degree values of the left middle temporal gyrus (MTG) and left fusiform gyrus (FFG).

				Unstandardized coefficients		Standardized coefficients	
Step		Δ*R^2^*	Adjusted *R^2^*	*B*	*SE*	*Beta*	*p*
1	Control variables	0.100	0.056				
	Sex			0.467	0.156	0.300	**0.004** ^**^
	Age			−0.273	0.171	−0.152	0.116
	Raven			0.001	0.031	0.003	0.973
2	Nodal degree values	0.190	0.243				
	Left MTG			0.304	0.063	0.512	< **0.001** ^***^
	Left FFG			0.209	0.101	0.216	**0.044** ^*^

Bold values indicate predictors whose p‐values are statistically significant (*p* < 0.05; two‐tailed) in the hierarchical linear regression model.

* *p* < 0.05

** *p* < 0.01

*** *p* < 0.001.

### 3.5 Mediation Analysis

**FIGURE 2:**
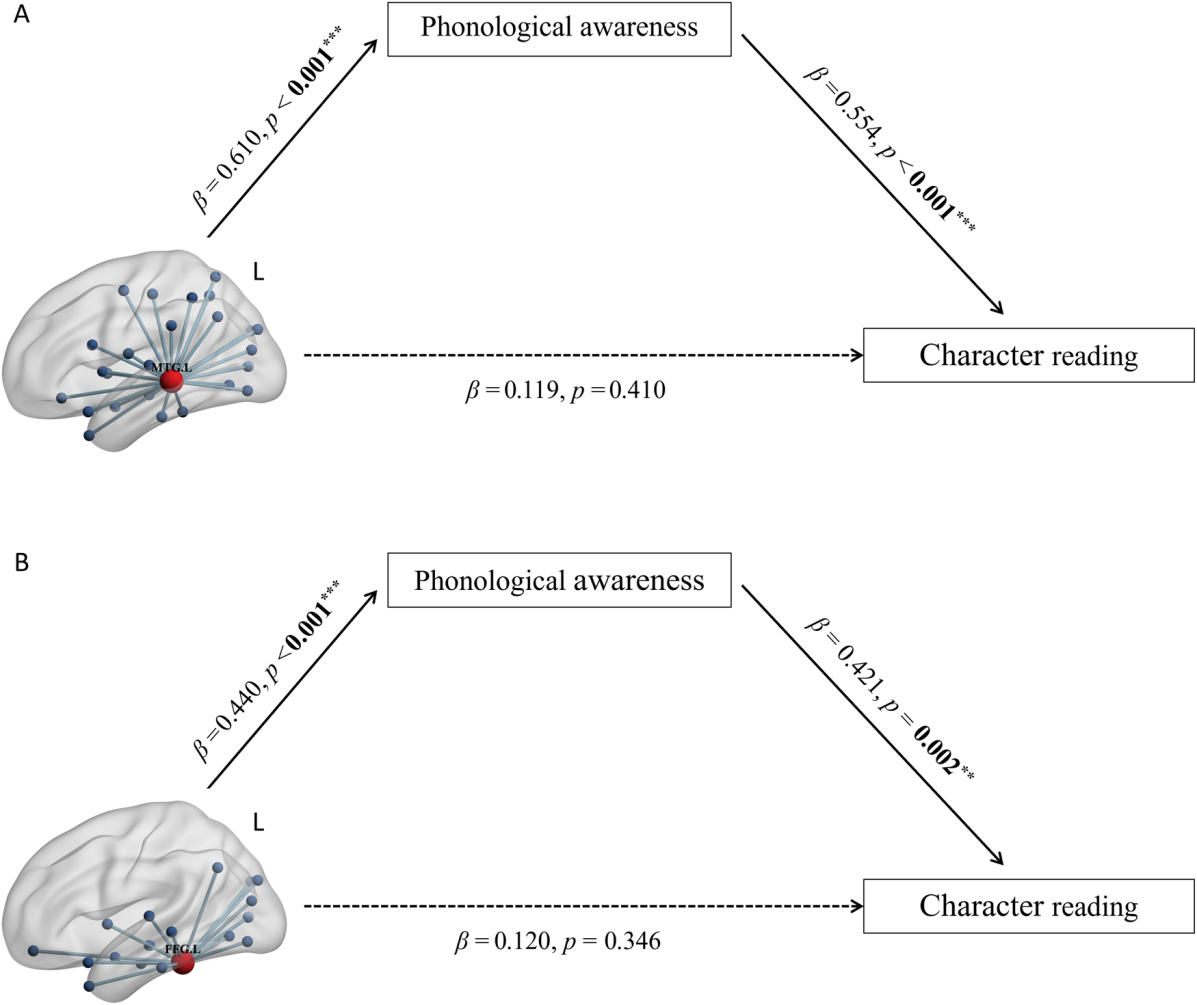
Top: The mediation model of white matter subnetwork around the left middle temporal gyrus (MTG), phonological awareness, and character reading. The associations between the left MTG and character reading are completely mediated by phonological awareness. Bottom: The mediation model of white matter subnetwork around the left fusiform gyrus (FFG), phonological awareness, and character reading. The associations between the left FFG and character reading are completely mediated by phonological awareness. All bold values represent the significance of *β* coefficients. ^**^ *p* < 0.01, ^***^ *p* < 0.001.

Two mediation models were tested, in which FA nodal degree values of the left MTG and the left FFG were independent variables, respectively. PA was the mediator, and character reading was the dependent variable. Age, sex, and IQ were covariates. Our results showed that the mediation effects of PA between the left MTG and FFG on character reading were both significant (left MTG: *β* = 0.338, 95% CI [0.135, 0.557]; left FFG: *β* = 0.185, 95% CI [0.050, 0.322]). In addition, the direct effect from the left MTG or FFG to character reading was not significant in the mediation models (left FFG: *β* = 0.120, 95% CI [−0.127, 0.367]; left MTG: *β* = 0.119, 95% CI [−0.406, 0.168]), suggesting full mediation effects of PA. In other words, the associations between the left MTG or the left FFG and character reading appeared to be  fully mediated by PA (see Figure 2).

## 4 Cross‐Modality Validation Analysis

To verify the robustness of the white matter network results, we further conducted cross‐modality validation analyses in the left MTG and FFG grey matter structure.

### 4.1 MRI Data Processing and Feature Extraction

FreeSurfer version 7.1.1 (Massachusetts General Hospital, Harvard Medical School; http://surfer.nmr.mgh.harvard.edu) was used to automatically segment neuroanatomical structures from T1‐weighted MRl scans. A series of steps, including the elimination of extraneous non‐brain matter, transformation into Talairach space, differentiation between gray and white matter, adjustment of intensity levels, and the application of both topological corrections and surface deformations to accurately delineate tissue boundaries, were processed (Dale et al. 1999).

Cortical thickness was defined by determining the minimal distance between the grey and white matter boundaries at each vertex over the cortex. Surface area was derived by computing the region between these two boundaries, and the local grey matter volume was estimated as the product of the calculated surface area and the corresponding thickness. Additionally, mean curvature was defined as the average of the two principal curvatures (calculated as the inverse of the radius of an inscribed circle), with higher values reflecting a more pronounced curvature. Cortical thickness, surface area, volume, and mean curvature were extracted for both the left MTG and the left FFG.

### 4.2 Partial Correlation and Mediation Analysis

Partial correlation analyses between grey matter structure measures of the left MTG/FFG and PA revealed only one significant result: a statistically significant positive correlation between the mean curvature of the left MTG and PA (*r* = 0.383, *p* = 0.003).

**FIGURE 3:**
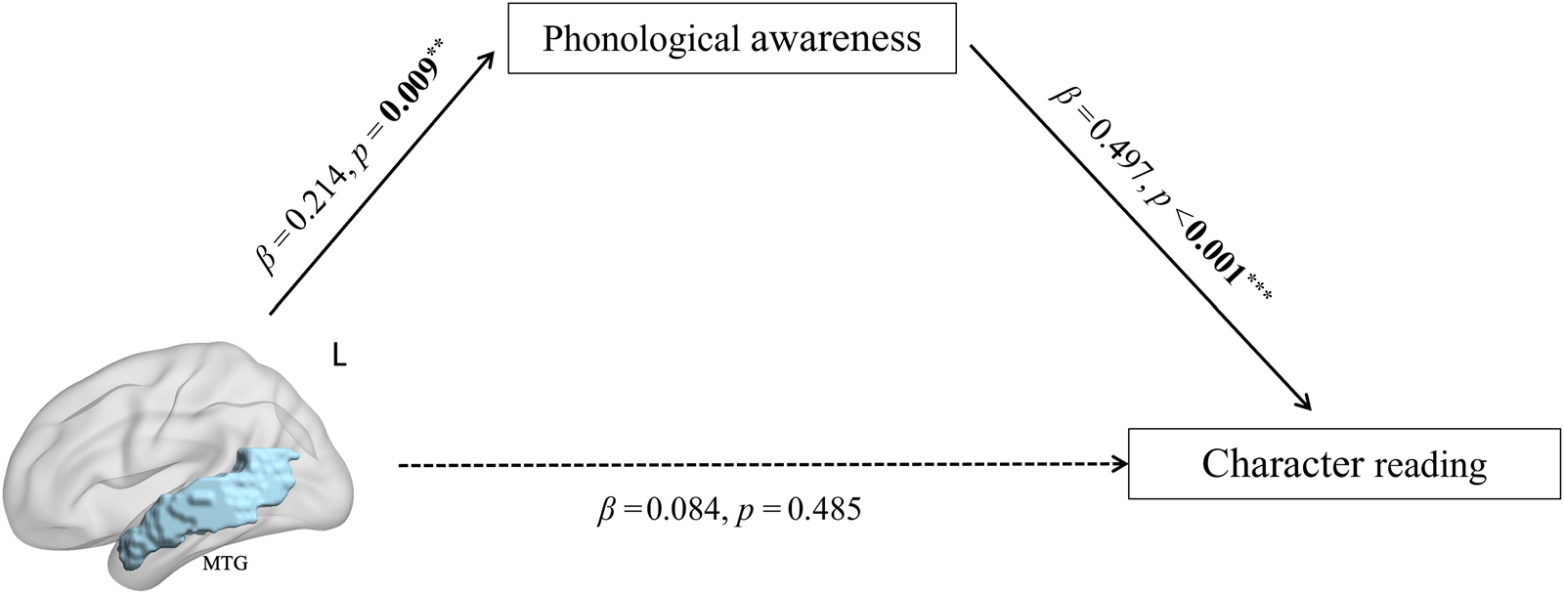
The mediation model of the left MTG mean curvature, phonological awareness, and character reading. The associations between the left MTG mean curvature and character reading are completely mediated by phonological awareness. All bold values represent the significance of *β* coefficients. ^**^ *p* < 0.01, ^***^ *p* < 0.001.

A mediation effect analysis was further conducted with the left MTG mean curvature as an independent variable, PA as mediator, and character reading as dependent variable. Age, sex, and IQ were covariates. We found that the indirect effect of the left MTG mean curvature on character reading was significant (*β* = 0.106, 95% CI [0.006, 0.218]). The direct effect from the left MTG mean curvature to character reading was not significant (*β* = 0.084, 95% CI [−0.317, 0.149]), suggesting a full mediation effect of PA. In other words, the association between the left MTG mean curvature and character reading was fully mediated by PA (see Figure 3).

## 5 Discussion

By employing a data‐driven, hub‐based white matter subnetwork method, we identified two distinct white matter subnetworks associated with PA in Chinese. One network was primarily centered on the left MTG, linking to subnetworks across the temporal, frontal, occipital, and parietal regions. Another network was focused around the left FFG, also linking to similar subnetworks across these brain regions. Both subnetworks were found to explain individual variations in PA and contributed to PA performance independently. Mediation analysis further demonstrated that PA fully mediated the relationship between these subnetworks and character reading ability in Chinese readers.

It is worth noting that in the ANCOVA analysis, although some brain regions showed trend‐level differences in nodal degree between poor and good readers, none survived after FDR correction. This result suggests that there may be no robust categorical differences in white matter networks between the two groups. This might be because the poor readers in our study were not clinically diagnosed with dyslexia but were individuals from the general population with relatively weaker reading abilities. These results align with previous research suggesting that reading ability and its neural correlates may vary along a continuum rather than reflect discrete categories (Shaywitz et al. 1992). To avoid overinterpreting nonsignificant group effects, the subsequent discussions were based on all the subjects.

First, the primary finding was the recognition of a white matter subnetwork centered on the left MTG corresponding to PA. This study demonstrated a significant correlation between PA and the subnetwork surrounding the left MTG in Chinese adults. The left MTG as the hub of the subnetwork was linked to the parietal, frontal, temporal, and occipital cortices. It specifically involved connections to regions such as the left ORBinf.L, SOG, MOG, SPG, STG, and ITG. The identification of this white matter subnetwork aligns with previously identified neural structures, indicating that reading and phonological processing may rely on the functional and structural connectivity of parietal and temporal networks (Hoeft et al. 2006; Boets et al. 2013; Vandermosten et al. 2012; Dębska, 2016; Centanni et al. 2019; Feng et al. 2023). These results corresponded with previous research conducted by our research team, which identified a similar white matter subnetwork centered on the left MTG linked to phonological processing in French children with dyslexia (Liu et al. 2022). Our findings provide further cross‐cultural evidence for white matter networks related to PA, suggesting that similar topological organizations support PA in nonalphabetic languages. Additionally, this study focused on the adult population, expanding on existing research that predominantly emphasized PA‐related white matter development in children. Given that white matter maturation is dynamically influenced by age (Hagmann et al. 2010), our results enhance the understanding of the white matter foundation of PA across different age groups. These results also align with a prior functional magnetic resonance imaging (fMRI) study conducted on Chinese adult readers, which indicated that the left MTG represented phonological information of character reading (Li et al. 2022). This research further verifies the essential role of the left MTG in phonological processing, complementing earlier findings based on white matter fiber tracts (Turker et al. 2023; Vandermosten et al. 2012; Saygin et al. 2013; Martins et al. 2024; Cao et al. 2017; Feng, 2020; Su et al. 2018). Although our study does not directly reconstruct white matter pathways, we hypothesize that the phonological processing subnetworks may involve the AF, ILF, part of the superior longitudinal fasciculus (SLF), and inferior fronto‐occipital fasciculus. Disruptions in these tracts have been associated with reduced phonological processing and reading performance in prior studies (Zhao et al. 2016; Lou et al. 2019; Su et al. 2018; Vandermosten et al. 2012; Yeatman et al. 2012). In sum, our study is in line with existing research and provides new insights into individual variations in brain–behavior relationships concerning PA and white matter subnetworks in Chinese adults.

Moreover, we found that PA completely mediated the relationship between the white matter subnetwork centered on the left MTG and character reading, shedding light on how the neural basis of PA influences character reading. This finding suggests a potential neural basis for character reading. To be specific, the white matter subnetwork centered on the left MTG is indirectly associated with character reading via PA, highlighting the mediating role of PA in linking the brain structure of left MTG to character reading (Koirala et al. 2021; Tang et al. 2024). This result supports the broad consensus that PA plays a critical role in reading abilities across various languages (Kovelman et al., 2012; Saygin et al., 2013; Shu et al. 2008; Wang et al. 2023). The neural mechanisms underlying PA and character reading in Chinese have previously been unclear, particularly in Chinese. Through mediation analysis, this study offers insight into how white matter connectivity of the left MTG relates to PA, which, in turn, is associated with character reading. Our research expands on earlier studies that primarily focused on alphabetic languages, offering a fresh perspective on the neural foundations of PA in Chinese reading. For the first time, we explored the mechanisms through which white matter networks influence character reading via PA. The left MTG not only plays a functional role in phonological processing (Li et al. 2022) but is also structurally connected to key white matter fiber tracts for phonological processing and reading, such as the AF, ILF, and SLF. While previous research suggests that deficits in the left MTG connectivity are often associated with reading difficulties, particularly in weakened phonological decoding abilities (Klingberg et al. 2000; Vandermosten et al. 2012), this study further illustrates the underying mechanism, that is, the left MTG, via its white matter connections with other critical brain regions, indirectly associates with reading performance through PA.

Our multimodal analysis provides additional support for the involvement of the left MTG in PA. It offers complementary evidence that PA mediates the relationship between structural properties of the left MTG and character reading performance. Consistent with the white matter subnetwork findings, a positive correlation was observed between PA and grey matter mean curvature in the left MTG. This cross‐modality analysis further highlights the importance of the left MTG in supporting PA, corroborating previous studies demonstrating that grey matter structure of the left temporal lobe, particularly the MTG, is closely associated with phonological processing and reading abilities (Richlan et al. 2013; Beyer et al. 2022; Vinckenbosch et al. 2005). While only the mean curvature of the left MTG showed a statistically significant association with PA, this result complemented the white matter findings in the same brain region. These results suggest that structural features of the left MTG across modalities may jointly reflect its involvement in phonological processing, although the specific mechanisms may differ. The grey matter result adds preliminary evidence to the role of left MTG and highlights the importance of multimodal approaches for understanding the neuroanatomical basis of PA.

Second, we found that the left FFG hub‐based white matter subnetwork was significantly associated with PA. This network comprised a hub in the left FFG that connected to the parietal, frontal, temporal, and occipital cortices, specifically encompassing the left ORBsup, SOG, MOG, TPOmid, STG, and ITG. Previous fMRI studies demonstrated that the phonetic information of Chinese characters was represented in the left FFG and adjacent areas (Li et al. 2022; Gu et al. 2024). Our research validates this finding and reveals that this ability is linked not only to the left FFG itself but also to its connectivity with other brain regions. These results suggest that the influence of the left FFG on reading abilities extends beyond visual information processing (Cohen et al., 2000; Cohen and Dehaene 2004; Dehaene, 2010; Liu et al. 2022) and further to phonological processing. In addition, PA fully mediated the relationship between the left FFG subnetwork and character reading. Consequently, this finding suggests that the left FFG plays a potential role in supporting phonological processing during character reading. Also, although the left FFG and MTG are both implicated in phonological processing, our collinearity diagnostics indicated low intercorrelation between these predictors (VIFs < 1.4; tolerance > 0.7). Our regression analysis further revealed that left FFG and MTG made unique contributions to the prediction of PA.

Additionally, our study observed sex differences in PA, consistent with previous findings, with female participants showing better performance (Chipere, 2013). This finding suggests that biological or sociocultural factors related to sex may contribute to individual differences in PA. Further research is needed to clarify the underlying mechanisms and whether they generalize across languages or developmental stages. Second, RAN has no significant difference between good and poor readers. This is consistent with our previous findings in Chinese adults with reading difficulties screened by C‐ARHQ (He et al. 2025). The result may be due to the RAN deficit being compensated in our poor readers. Alternatively, it may be because the RAN deficit was considered a core feature of developmental dyslexia (Shum and Au 2016; Wolf and Bowers 1999), whereas the poor readers in our study were not clinically diagnosed as dyslexics. RAN was also not significantly associated with any brain regions’ nodal degree in our study. This result aligned with our previous findings, which similarly reported no significant correlations between RAN and white matter network parameters (Lou et al. 2019) and nodal degree values of white matter connectivity (Liu et al. 2022). Although earlier research has identified associations between RAN and the integrity of specific white matter fiber tracts, such as the AF (Cross et al. 2023), RAN may be more closely related to microstructural properties of white matter fiber tracts rather than topological features at the nodal level.

It should be acknowledged that the present study included several potential limitations. First, our participants were normal adults from colleges with similar educational backgrounds, resulting in potentially smaller variability among subjects. Second, our study suggested that while the backbone network defined by good readers was stable and reliable for the main results, subtle differences might arise when defining the backbone network based on all participants, particularly in the left FFG (see the Supporting Information). These differences have minimal impact on the findings of our study, but they emphasize the need for considering broader group definitions or comparing multiple backbone network definitions in future research to ensure the robustness of the results. Furthermore, while our findings are consistent across languages and age groups in white matter networks associated with PA, the relationship between the brain plasticity of these networks and the longitudinal dynamics of PA remains unclear. Future longitudinal studies may offer a deeper understanding of the evolving brain mechanisms of white matter networks underlying PA during development.

In conclusion, this study identified the white matter networks corresponding to PA through a data‐driven hub‐based network analysis. The left MTG and FFG hub‐based white matter subnetworks were substantially linked with PA in Chinese readers. The findings are largely consistent with our previous study on French children (Liu et al. 2022). Furthermore, the study revealed that PA fully mediates the relationship between the white matter subnetworks based on the left MTG and FFG and character reading. These findings provide the first anatomical evidence of white matter subnetworks uniquely associated with PA in Chinese adults. The results offer an important cross‐linguistic insight into the white matter network corresponding to PA in nonalphabetic languages such as Chinese. Future longitudinal and interventional studies are needed to clarify the extent to which these features may serve as predictors or clinical indicators of reading difficulties.

## Author Contributions

**Xinyue Zhang**: data curation, formal analysis, methodology, visualization, writing – original draft, writing – review and editing. **Yueye Zhao**: data curation, formal analysis, investigation, methodology, writing – review and editing. **Siyu Chen**: data curation, formal analysis, methodology, writing – review and editing. **Zi‐gang Huang**: funding acquisition, resources. **Jingjing Zhao**: conceptualization, data curation, methodology, project administration, resources, supervision, writing – review and editing.

## Conflicts of Interest

The authors declare no conflicts of interest.

## Ethics Statement

The Shaanxi Normal University Ethics Committee approved the study (HRHR2024‐04‐01). All subjects provided written informed consent prior to MRI and behavioural investigation.

## Peer Review

The peer review history for this article is available at https://publons.com/publon/10.1002/brb3.70781

## Supporting information




**Supporting Table S1**: brb370781‐sup‐0001‐tableS1.docx

**Supporting Table S2**: brb370781‐sup‐0002‐tableS2.docx

**Supplementary information**: brb370781‐sup‐0003‐SuppMat.docx

## Data Availability

Data will be made available on reasonable request.

## References

[brb370781-bib-0001] Alexander, A. L. , J. E. Lee , M. Lazar , and A. S. Field . 2007. “Diffusion Tensor Imaging of the Brain.” Neurotherapeutics 4, no. 3: 316–329. 10.1016/j.nurt.2007.05.011. 17599699 PMC2041910

[brb370781-bib-0002] Bassett, D. S. , and E. T. Bullmore . 2009. “Human Brain Networks in Health and Disease.” Current Opinion in Neurology 22, no. 4: 340–347. 10.1097/WCO.0b013e32832d93dd. 19494774 PMC2902726

[brb370781-bib-0003] Beaulieu, C. 2002. “The Basis of Anisotropic Water Diffusion in the Nervous System—A Technical Review.” NMR in Biomedicine 15, no. 7–8: 435–455. 10.1002/nbm.782. 12489094

[brb370781-bib-0004] Benjamini, Y. , and Y. Hochberg . 1995. “Controlling the False Discovery Rate: A Practical and Powerful Approach to Multiple Hypothesis Testing.” Journal of the Royal Statistical Society: Series B 57: 289–300. 10.1111/j.2517-6161.1995.tb02031.x.

[brb370781-bib-0005] Beyer, M. , J. Liebig , T. Sylvester , et al. 2022. “Structural Gray Matter Features and Behavioral Preliterate Skills Predict Future Literacy—A Machine Learning Approach.” Frontiers in Neuroscience 16: 920150. 10.3389/fnins.2022.920150. 36248649 PMC9558903

[brb370781-bib-0006] Boets, B. , H. P. O. de Beeck , M. Vandermosten , et al. 2013. “Intact but Less Accessible Phonetic Representations in Adults With Dyslexia.” Science 342, no. 6163: 1251–1254. 10.1126/science.1244333. 24311693 PMC3932003

[brb370781-bib-0007] Bradley, L. , and P. E. Bryant . 1985. Rhyme and Reason in Reading and Spelling. University of Michigan Press.

[brb370781-bib-0008] Bullmore, E. T. , and D. S. Bassett . 2011. “Brain Graphs: Graphical Models of the Human Brain Connectome.” Annual Review of Clinical Psychology 7, no. 1: 113–140. 10.1146/annurev-clinpsy-040510-143934. 21128784

[brb370781-bib-0009] Cao, F. , X. Yan , Z. Wang , et al. 2017. “Neural Signatures of Phonological Deficits in Chinese Developmental Dyslexia.” Neuroimage 146: 301–311. 10.1016/j.neuroimage.2016.11.051. 27890803

[brb370781-bib-0010] Cao, F. , X. Yan , X. Yan , H. Zhou , and J. R. Booth . 2020. “Reading Disability in Chinese Children Learning English as an L2.” Child Development 13452: e126–e142. 10.1111/cdev.13452. 32864778

[brb370781-bib-0011] Castles, A. , and M. Coltheart . 2004. “Is There a Causal Link From Phonological Awareness to Success in Learning to Read?” Cognition 91, no. 1: 77–111. 10.1016/S0010-0277(03)00164-1. 14711492

[brb370781-bib-0012] Centanni, T. M. , E. S. Norton , O. Ozernov‐Palchik , et al. 2019. “Disrupted Left Fusiform Response to Print in Beginning Kindergartners Is Associated With Subsequent Reading.” NeuroImage. Clinical 22: 101715. 10.1016/j.nicl.2019.101715. 30798165 PMC6389729

[brb370781-bib-0013] Cheng, C. , Y. Yao , Z. Wang , and J. Zhao . 2021. “Visual Attention Span and Phonological Skills in Chinese Developmental Dyslexia.” Research in Developmental Disabilities 116: 104015. 10.1016/j.ridd.2021.104015. 34182333

[brb370781-bib-0014] Chipere, N. 2013. “Sex Differences in Phonological Awareness and Reading Ability.” Language Awareness 23, no. 3: 275–289. 10.1080/09658416.2013.774007.

[brb370781-bib-0015] Cohen, L. , and S. Dehaene . 2004. “Specialization Within the Ventral Stream: The Case for the Visual Word Form Area.” Neuroimage 22, no. 1: 466–476. 10.1016/j.neuroimage.2003.12.049. 15110040

[brb370781-bib-0016] Cohen, L. , S. Dehaene , L. Naccache , et al. 2000. “The Visual Word Form Area: Spatial and Temporal Characterization of an Initial Stage of Reading in Normal Subjects and Posterior Split‐brain Patients.” Brain 123, no. Pt2: 291–307. 10.1093/brain/123.2.291. 10648437

[brb370781-bib-0017] Cross, A. M. , J. M. Lammert , L. Peters , et al. 2023. “White Matter Correlates of Reading Subskills in Children With and Without Reading Disability.” Brain and Language 241: 105270. 10.1016/j.bandl.2023.105270. 37141728

[brb370781-bib-0018] Cui, Z. , S. Zhong , P. Xu , Y. He , and G. Gong . 2013. “PANDA: A Pipeline Toolbox for Analyzing Brain Diffusion Images.” Frontiers in Human Neuroscience 7: 42. 10.3389/fnhum.2013.00042. 23439846 PMC3578208

[brb370781-bib-0019] Dale, A. M. , B. Fischl , and M. I. Sereno . 1999. “Cortical Surface‐Based Analysis. I. Segmentation and Surface Reconstruction.” Neuroimage 9: 179–194. 10.1006/nimg.1998.0395. 9931268

[brb370781-bib-0020] Dębska, A. , M. Łuniewska , K. Chyl , et al. 2016. “Neural Basis of Phonological Awareness in Beginning Readers With Familial Risk of Dyslexia‐Results From Shallow Orthography.” Neuroimage 132: 406–416. 10.1016/j.neuroimage.2016.02.063. 26931814

[brb370781-bib-0021] Dehaene, S. 2010. Reading in the Brain: The New Science of How We Read. Penguin Group USA.

[brb370781-bib-0022] Denckla, M. B. , and R. G. Rudel . 1976. “Rapid Automatized Naming (RAN): Dyslexia Differentiated From Other Learning Disabilities.” Neuropsychologia 14, no. 4: 471–479. 10.1016/0028-3932(76)90075-0. 995240

[brb370781-bib-0023] Dennis, E. L. , N. Jahanshad , A. W. Toga , et al. 2012. “Test‐Retest Reliability of Graph Theory Measures of Structural Brain Connectivity.” In Medical Image Computing and Computer‐Assisted Intervention (MICCAI2012): International Conference on Medical Image Computing and Computer‐Assisted Intervention, 305–312. Lecture Notes in Computer Science, volume 7512. Springer. 10.1007/978-3-642-33454-2_38. PMC4039303 23286144

[brb370781-bib-0024] Deutsch, G. K. , R. F. Dougherty , R. Bammer , W. T. Siok , J. D. E. Gabrieli , and B. Wandell . 2005. “Children's Reading Performance Is Correlated With White Matter Structure Measured by Tensor Imaging.” Cortex; A Journal Devoted to the Study of the Nervous System and Behavior 41: 354–363. 10.1016/s0010-9452(08)70272-7. 15871600

[brb370781-bib-0025] de Vos, A. , J. Vanderauwera , S. Vanvooren , M. Vandermosten , P. Ghesquière , and J. Wouters . 2020. “The Relation Between Neurofunctional and Neurostructural Determinants of Phonological Processing in Pre‐readers.” Developmental Cognitive Neuroscience 46: 100874. 10.1016/j.dcn.2020.100874. 33130464 PMC7606842

[brb370781-bib-0026] Ding, N. , P. Peng , S. Li , J. Tang , and J. Zhao . 2024. “Profiles of Phonological Deficits and Comorbidity in Chinese Developmental Dyslexia.” *Reading and Writing*. Ahead of print. 10.1007/s11145-024-10615-7.

[brb370781-bib-0027] Ding, N. , P. Peng , J. Tang , Y. Ding , and J. Zhao . 2025. “An Investigation of Phonological Skills in Chinese Developmental Dyslexia.” *Reading and Writing*. Ahead of print. 10.1007/s11145-024-10618-4.

[brb370781-bib-0028] Feng, G. , X. Yan , L. Shen , et al. 2023. “Distinct Neural Correlates of Poor Decoding and Poor Comprehension in Children With Reading Disability.” Cerebral Cortex 33, no. 6: 3239–3254. 10.1093/cercor/bhac272. 35848850

[brb370781-bib-0029] Feng, X. , I. Altarelli , K. Monzalvo , et al. 2020. “A Universal Reading Network and Its Modulation by Writing System and Reading Ability in French and Chinese Children.” Elife 9: e54591. 10.7554/eLife.54591. 33118931 PMC7669264

[brb370781-bib-0030] Gong, G. , Y. He , L. Concha , et al. 2009. “Mapping Anatomical Connectivity Patterns of Human Cerebral Cortex Using in Vivo Diffusion Tensor Imaging Tractography.” Cerebral Cortex 19, no. 3: 524–536. 10.1093/cercor/bhn102. 18567609 PMC2722790

[brb370781-bib-0031] Gu, L. , Y. Pang , J. Yang , J. Qu , N. Gu , and L. Mei . 2024. “Orthographic and Phonological Processing in the Left Ventral Occipitotemporal Cortex During Chinese Word Reading.” Psychophysiology 61: e14703. 10.1111/psyp.14703. 39367529

[brb370781-bib-0032] Hagmann, P. , O. Sporns , N. Madan , et al. 2010. “White Matter Maturation Reshapes Structural Connectivity in the Late Developing Human Brain.” Proceedings of the National Academy of Sciences 107, no. 44: 19067–19072. 10.1073/pnas.1009073107. PMC2973853 20956328

[brb370781-bib-0033] He, Y. , J. Tang , X. Yang , et al. 2025. “Development and Validity of the Adult Reading History Questionnaire (ARHQ) for Chinese.” Dyslexia 31: e1802. 10.1002/dys.1802. 39907021 PMC11795345

[brb370781-bib-0034] Hoeft, F. , A. Hernandez , G. McMillon , et al. 2006. “Neural Basis of Dyslexia: A Comparison Between Dyslexic and Nondyslexic Children Equated for Reading Ability.” Journal of Neuroscience 26: 10700–10708. 10.1523/JNEUROSCI.4931-05.2006. 17050709 PMC6674758

[brb370781-bib-0035] Hulme, C. , M. Snowling , M. Caravolas , and J. Carroll . 2005. “Phonological Skills Are (Probably) One Cause of Success in Learning to Read: A Comment on Castles and Coltheart.” Scientific Studies of Reading 9, no. 4: 351–365. 10.1207/s1532799xssr0904_2.

[brb370781-bib-0036] Jones, D. K. , T. R. Knösche , and R. Turner . 2013. “White Matter Integrity, Fiber Count, and Other Fallacies: The Do's and Don'ts of Diffusion MRI.” Neuroimage 73: 239–254. 10.1016/j.neuroimage.2012.06.081. 22846632

[brb370781-bib-0037] Keller, T. A. , and M. A. Just . 2009. “Altering Cortical Connectivity: Remediation‐Induced Changes in the White Matter of Poor Readers.” Neuron 64, no. 5: 624–631. 10.1016/j.neuron.2009.10.018. 20005820 PMC2796260

[brb370781-bib-0038] Klingberg, T. , M. Hedehus , E. Temple , T. Salz , J. D. Gabrieli , et al. 2000. “Microstructure of Temporoparietal White Matter as a Basis for Reading Ability: Evidence From Diffusion Tensor Magnetic Resonance Imaging.” Neuron 25: 493–500. 10.1016/s0896-6273(00)80911-3. 10719902

[brb370781-bib-0039] Koirala, N. , M. V. Perdue , X. Su , E. L. Grigorenko , and N. Landi . 2021. “Neurite Density and Arborization Is Associated With Reading Skill and Phonological Processing in Children.” Neuroimage 241: 118426. 10.1016/j.neuroimage.2021.118426. 34303796 PMC8539928

[brb370781-bib-0040] Kovelman, I. , E. S. Norton , J. A. Christodoulou , et al. 2012. “Brain Basis of Phonological Awareness for Spoken Language in Children and Its Disruption in Dyslexia.” Cerebral Cortex 22, no. 4: 754–764. 10.1093/cercor/bhr094. 21693783 PMC4498147

[brb370781-bib-0041] Landerl, K. , F. Ramus , K. Moll , et al. 2013. “Predictors of Developmental Dyslexia in European Orthographies With Varying Complexity.” Journal of Child Psychology and Psychiatry 54, no. 6: 686–694. 10.1111/jcpp.12029. 23227813

[brb370781-bib-0042] Langer, N. , B. Peysakhovich , J. Zuk , et al. 2017. “White Matter Alterations in Infants at Risk for Developmental Dyslexia.” Cerebral Cortex 27, no. 2: 1027–1036. 10.1093/cercor/bhv281. 26643353 PMC6074795

[brb370781-bib-0043] Li, A. , R. Yang , J. Qu , J. Dong , L. Gu , and L. Mei . 2022. “Neural Representation of Phonological Information During Chinese Character Reading.” Human Brain Mapping 43, no. 13: 4013–4029. 10.1002/hbm.25900. 35545935 PMC9374885

[brb370781-bib-0044] Liu, T. , M. Thiebaut de Schotten , I. Altarelli , F. Ramus , and J. Zhao . 2021. “Maladaptive Compensation of Right Fusiform Gyrus in Developmental Dyslexia: A Hub‐based White Matter Network Analysis.” Cortex; A Journal Devoted to the Study of the Nervous System and Behavior 145: 57–66. 10.1016/j.cortex.2021.07.016. 34689032

[brb370781-bib-0045] Liu, T. , M. Thiebaut de Schotten , I. Altarelli , F. Ramus , and J. Zhao . 2022. “Neural Dissociation of Visual Attention Span and Phonological Deficits in Developmental Dyslexia: A Hub‐based White Matter Network Analysis.” Human Brain Mapping 43, no. 17: 5210–5219. 10.1002/hbm.25997. 35808916 PMC9812243

[brb370781-bib-0046] Lou, C. , X. Duan , I. Altarelli , J. A. Sweeney , F. Ramus , and J. Zhao . 2019. “White Matter Network Connectivity Deficits in Developmental Dyslexia.” Human Brain Mapping 40, no. 2: 505e516. 10.1002/hbm.24390. 30251768 PMC6865529

[brb370781-bib-0047] MacKinnon, D. P. , C. M. Lockwood , and J. Williams . 2004. “Confidence Limits for the Indirect Effect: Distribution of the Product and Resampling Methods.” Multivariate Behavioral Research 39, no. 1: 99–128. 10.1207/s15327906mbr3901_4. 20157642 PMC2821115

[brb370781-bib-0048] Martins, B. , M. Y. Baba , E. M. Dimateo , et al. 2024. “Investigating Dyslexia Through Diffusion Tensor Imaging Across Ages: A Systematic Review.” Brian Sciences 14, no. 4: 349. 10.3390/brainsci14040349. PMC11047980 38672001

[brb370781-bib-0049] McCartney, K. , M. R. Burchinal , and K. L. Bub . 2006. “Best Practices in Quantitative Methods for Developmentalists.” Research in Child Development 71, no. 3: 1–145. 10.1111/j.1540-5834.2006.07103001.x. 17199773

[brb370781-bib-0050] Newman, E. H. , T. Tardif , J. Huang , and H. Shu . 2011. “Phonemes Matter: The Role of Phoneme‐Level Awareness in Emergent Chinese Readers.” Journal of Experimental Child Psychology 108: 242–259. 10.1016/j.jecp.2010.09.001. 20980019 PMC3644705

[brb370781-bib-0051] Norton, E. S. , and M. Wolf . 2012. “Rapid Automatized Naming (RAN) and Reading Fluency: Implications for Understanding and Treatment of Reading Disabilities.” Annual Review of Psychology 63: 427–452. 10.1146/annurev-psych-120710-100431. 21838545

[brb370781-bib-0052] Pan, J. , S. Song , M. Su , et al. 2016. “On the Relationship Between Phonological Awareness, Morphological Awareness and Chinese Literacy Skills: Evidence From an 8‐year Longitudinal Study.” Developmental Science 19, no. 6: 982–991. 10.1111/desc.12356. 26537834

[brb370781-bib-0053] Ramus, F. , I. Altarelli , K. Jednoróg , J. Zhao , and L. Scotto di Covella . 2018. “Neuroanatomy of Developmental Dyslexia: Pitfalls and Promise.” Neuroscience & Biobehavioral Reviews 84: 434–452. 10.1016/j.neubiorev.2017.08.001. 28797557

[brb370781-bib-0054] Ramus, F. , and G. Szenkovits . 2008. “What Phonological Deficit?” The Quarterly Journal of Experimental Psychology 61, no. 1: 129–141. 10.1080/17470210701508822. 18038344

[brb370781-bib-0055] Reynolds, J. E. , X. Long , M. N. Grohs , D. Dewey , and C. Lebel . 2019. “Structural and Functional Asymmetry of the Language Network Emerge in Early Childhood.” Development Cognitive Neuroscience 39: 100682. 10.1016/j.dcn.2019.100682. PMC6969356 31376589

[brb370781-bib-0056] Richlan, F. , M. Kronbichler , and H. Wimmer . 2013. “Structural Abnormalities in the Dyslexic Brain: A Meta‐Analysis of Voxel‐Based Morphometry Studies.” Human Brain Mapping 34, no. 11: 3055–3065. 10.1002/hbm.22127. 22711189 PMC6870307

[brb370781-bib-0057] Rimrodt, S. L. , D. J. Peterson , M. B. Denckla , W. E. Kaufmann , and L. E. Cutting . 2010. “White Matter Microstructural Differences Linked to Left Perisylvian Language Network in Children With Dyslexia.” Cortex; A Journal Devoted to the Study of the Nervous System and Behavior 46, no. 6: 739–749. 10.1016/j.cortex.2009.07.008. 19682675 PMC2847658

[brb370781-bib-0058] Saksida, A. , S. Iannuzzi , C. Bogliotti , et al. 2016. “Phonological Skills, Visual Attention Span, and Visual Stress in Developmental Dyslexia.” Developmental Psychology 52, no. 10: 1503–1516. 10.1037/dev0000184. 27690491

[brb370781-bib-0059] Saygin, Z. M. , E. S. Norton , D. E. Osher , et al. 2013. “Tracking the Roots of Reading Ability: White Matter Volume and Integrity Correlate With Phonological Awareness in Prereading and Early‐reading Kindergarten Children.” Journal of Neuroscience 33, no. 33: 13251–13258. 10.1523/JNEUROSCI.4383-12.2013. 23946384 PMC3742917

[brb370781-bib-0060] Shaywitz, S. E. , M. D. Escobar , B. A. Shaywitz , J. M. Fletcher , and R. Makuch . 1992. “Evidence That Dyslexia May Represent the Lower Tail of a Normal Distribution of Reading Ability.” The New England Journal of Medicine 326, no. 3: 145–150. 10.1056/NEJM199201163260301. 1727544

[brb370781-bib-0061] Shu, H. , C. McBride‐Chang , S. Wu , and H. Liu . 2006. “Understanding Chinese Developmental Dyslexia: Morphological Awareness as a Core Cognitive Construct.” Journal of Educational Psychology 98, no. 1: 122–133. 10.1037/0022-0663.98.1.122.

[brb370781-bib-0062] Shu, H. , H. Peng , and C. McBride‐Chang . 2008. “Phonological Awareness in Young Chinese Children.” Developmental Science 11, no. 1: 171–181. 10.1111/j.1467-7687.2007.00654.x. 18171377

[brb370781-bib-0063] Shum, K. K. M. , and T. K. F. Au . 2016. “Why Does Rapid Naming Predict Chinese Word Reading?” Language Learning and Development 13, no. 1: 127–142. 10.1080/15475441.2016.1232651.

[brb370781-bib-0064] Snowling, M. J. , and M. Melby‐Lervåg . 2016. “Oral Language Deficits in Familial Dyslexia: A Meta‐Analysis and Review.” Psychological Bulletin 142, no. 5: 498–545. 10.1037/bul0000037. 26727308 PMC4824243

[brb370781-bib-0065] Sotiropoulos, S. N. , and A. Zalesky . 2019. “Building Connectomes Using Diffusion MRI: Why, How and But.” NMR in Biomedicine 32, no. 4: e3752. 10.1002/nbm.3752. 28654718 PMC6491971

[brb370781-bib-0066] Su, M. , J. Zhao , M. Thiebaut de Schotten , et al. 2018. “Alterations in White Matter Pathways Underlying Phonological and Morphological Processing in Chinese Developmental Dyslexia.” Developmental Cognitive Neuroscience 31: 11–19. 10.1016/j.dcn.2018.04.002. 29727819 PMC6969203

[brb370781-bib-0067] Tang, X. , T. K. Turesky , E. S. Escalante , et al. 2024. “Longitudinal Associations Between Language Network Characteristics in the Infant Brain and School‐Age Reading Abilities Are Mediated by Early‐Developing Phonological Skills.” Developmental Cognitive Neuroscience 68: 101405. 10.1016/j.dcn.2024.101405. 38875769 PMC11225703

[brb370781-bib-0068] Tong, X. , C. Mcbride‐Chang , A. M. Y. Wong , H. Shu , P. Reitsma , and J. Rispens . 2011. “Longitudinal Predictors of Very Early Chinese Literacy Acquisition.” Journal of Research in Reading 34, no. 3: 315–332. 10.1111/j.1467-9817.2009.01426.x.

[brb370781-bib-0069] Turker, S. , P. Kuhnke , S. B. Eickhoff , S. Caspers , and G. Hartwigsen . 2023. “Cortical, Subcortical, and Cerebellar Contributions to Language Processing: A Meta‐Analytic Review of 403 Neuroimaging Experiments.” Psychological Bulletin 149, no. 11–12: 699–723. 10.1037/bul0000403. 37768610

[brb370781-bib-0070] Tzourio‐Mazoyer, N. , B. Landeau , D. Papathanassiou , et al. 2002. “Automated Anatomical Labeling of Activations in SPM Using a Macroscopic Anatomical Parcellation of the MNI MRI Single‐subject Brain.” Neuroimage 15, no. 1: 273–289. 10.1006/nimg.2001.0978. 11771995

[brb370781-bib-0071] van den Heuvel, M. P. , and O. Sporns . 2013. “Network Hubs in the Human Brain.” Trends in Cognitive Sciences 17, no. 12: 683–696. 10.1016/j.tics.2013.09.012. 24231140

[brb370781-bib-0072] Vanderauwera, J. , M. Vandermosten , F. Dell'Acqua , J. Wouters , and P. Ghesquière . 2015. “Disentangling the Relation Between Left Temporoparietal White Matter and Reading: A Spherical Deconvolution Tractography Study.” Human Brain Mapping 36, no. 8: 3273–3287. 10.1002/hbm.22848. 26037303 PMC6869773

[brb370781-bib-0073] Vandermosten, M. , B. Boets , H. Poelmans , S. Sunaert , J. Wouters , and P. Ghesquiere . 2012. “A Tractography Study in Dyslexia: Neuroanatomic Correlates of Orthographic, Phonological and Speech Processing.” Brain 135, no. 3: 935–948. 10.1093/brain/awr363. 22327793

[brb370781-bib-0074] Vandermosten, M. , J. Vanderauwera , C. Theys , et al. 2015. “A DTI Tractography Study in Pre‐Readers at Risk for Dyslexia.” Developmental Cognitive Neuroscience 14: 8–15. 10.1016/j.dcn.2015.05.006. 26048528 PMC6989819

[brb370781-bib-0075] Vinckenbosch, E. , F. Robichon , and S. Eliez . 2005. “Gray Matter Alteration in Dyslexia: Converging Evidence From Volumetric and Voxel‐by‐Voxel MRI Analyses.” Neuropsychologia 43, no. 3: 324–331. 10.1016/j.neuropsychologia.2004.06.023. 15707610

[brb370781-bib-0076] Wang, Z. , Y. Yu , Y. Xia , et al. 2023. “The Effects of Phonological Awareness and Morphological Awareness on Reading Comprehension in Early Elementary School Children: The Mediating Role of Reading Fluency.” Acta Psychologica Sinica 55, no. 6: 930–940. 10.3724/SP.J.1041.2023.00930.

[brb370781-bib-0084] Wolf, M. , A. G. O'Rourke , C. Gidney , M. Lovett , P. Cirino , and R. Morris . 2002. “The Second Deficit: An Investigation of the Independence of Phonological and Naming‐Speed Deficits in Developmental Dyslexia.” Reading and Writing: An Interdisciplinary Journal 15, no. 1–2: 43–72. 10.1023/A:1013816320290.

[brb370781-bib-0077] Wolf, M. , and P. G. Bowers . 1999. “The Double‐Deficit Hypothesis for the Developmental Dyslexias.” Journal of Educational Psychology 91, no. 3: 415–438. 10.1037/0022-0663.91.3.415.

[brb370781-bib-0078] Yeatman, J. D. , R. F. Dougherty , M. Ben‐Shachar , and B. A. Wandell . 2012. “Development of White Matter and Reading Skills.” Proceedings of the National Academy of Sciences 109, no. 44: E3045–E3053. 10.1073/pnas.1206792109 PMC3497768 23045658

[brb370781-bib-0079] Yeatman, J. D. , R. F. Dougherty , E. Rykhlevskaia , et al. 2011. “Anatomical Properties of the Arcuate Fasciculus Predict Phonological and Reading Skills in Children.” Journal of Cognitive Neuroscience 23, no. 11: 3304–3317. 10.1162/jocn_a_00061. 21568636 PMC3214008

[brb370781-bib-0080] Zhao, J. , M. Thiebaut de Schotten , I. Altarelli , J. Dubois , and F. Ramus . 2016. “Altered Hemispheric Lateralization of White Matter Pathways in Developmental Dyslexia: Evidence From Spherical Deconvolution Tractography.” Cortex; A Journal Devoted to the Study of the Nervous System and Behavior 76: 51–62. 10.1016/j.cortex.2015.12.004. 26859852

[brb370781-bib-0082] Zhao, Y. , J. Liu , X. Ma , Z. G. Huang , and J. Zhao . 2025. “Microstructural Lateralization of Thalamocortical Connections in Individuals With a History of Reading Difficulties.” Neuroimage 308: 121071. 10.1016/j.neuroimage.2025.121071. 39894236

[brb370781-bib-0083] Ziegler, J. C. , and U. Goswami . 2005. “Reading Acquisition, Developmental Dyslexia, and Skilled Reading Across Languages: A Psycholinguistic Grain Size Theory.” Psychology Bulletin 131: 3–29. 10.1037/0033-2909.131.1.3. 15631549

