# Supplementary material for: White Matter Networks of Phonological Awareness in Chinese Readers
Source: Brain Behav. 2025 Sep 21;15(9):e70781. doi: 10.1002/brb3.70781 (PMC12451024; doi:10.1002/brb3.70781)
Supplement: Supplementary file 1 — Supporting Table S1: brb370781‐sup‐0001‐tableS1.docx [file BRB3-15-e70781-s002.docx]

Table S1: The analysis of covariance results of of nodal degree values’ group differences in white matter.

| Nodal degree values | | Mean squared | F | p |
| --- | --- | --- | --- | --- |
| PreCG.L | Precental gyrus | 1.375 | 1.036 | 0.396 |
| PreCG.R | Precental gyrus | 1.029 | 0.694 | 0.599 |
| SFGdor.L | Superior frontal gyrus, dorsolateral | 0.644 | 0.488 | 0.744 |
| SFGdor.R | Superior frontal gyrus, dorsolateral | 1.028 | 0.641 | 0.635 |
| ORBsup.L | Superior frontal gyrus, orbital part | 1.082 | 0.876 | 0.484 |
| ORBsup.R | Superior frontal gyrus, orbital part | 3.034 | 1.269 | 0.292 |
| MFG.L | Middle frontal gyrus | 0.960 | 3.269 | **0.017** |
| MFG.R | Middle frontal gyrus | 0.634 | 1.243 | 0.302 |
| ORBmid.L | Middle frontal gyrus, orbital part | 0.314 | 0.344 | 0.847 |
| ORBmid.R | Middle frontal gyrus, orbital part | 0.588 | 0.655 | 0.626 |
| IFGoperc.L | Inferior frontal gyrus, opercular part | 1.041 | 1.387 | 0.249 |
| IFGoperc.R | Inferior frontal gyrus, opercular part | 1.799 | 2.245 | 0.075 |
| IFGtriang.L | Inferior frontal gyrus, triangular part | 0.369 | 0.218 | 0.928 |
| IFGtriang.R | Inferior frontal gyrus, triangular part | 4.729 | 2.414 | 0.059 |
| ORBinf.L | Inferior frontal gyrus, orbital part | 1.543 | 1.010 | 0.410 |
| ORBinf.R | Inferior frontal gyrus, orbital part | 3.645 | 1.951 | 0.114 |
| ROL.L | Rolandic operculum | 0.952 | 2.399 | 0.060 |
| ROL.R | Rolandic operculum | 1.601 | 3.115 | **0.021** |
| SMA.L | Supplementary motor area | 2.423 | 1.581 | 0.191 |
| SMA.R | Supplementary motor area | 1.668 | 1.284 | 0.286 |
| OLF.L | Olfactory cortex | 0.396 | 0.265 | 0.899 |
| OLF.R | Olfactory cortex | 0.844 | 0.603 | 0.662 |
| SFGmed.L | Superior frontal gyrus, medial | 2.732 | 1.722 | 0.157 |
| SFGmed.R | Superior frontal gyrus, medial | 2.839 | 2.415 | 0.059 |
| ORBsupmed.L | Superior frontal gyrus, medial orbital | 4.355 | 3.703 | **0.009** |
| ORBsupmed.R | Superior frontal gyrus, medial orbital | 1.440 | 1.110 | 0.360 |
| REC.L | Gyrus rectus | 3.107 | 2.736 | **0.037** |
| REC.R | Gyrus rectus | 1.336 | 2.178 | 0.082 |
| INS.L | Insula | 0.527 | 0.656 | 0.625 |
| INS.R | Insula | 1.750 | 1.058 | 0.385 |
| ACG.L | Anterior cingulate and paracingulate gyri | 0.274 | 0.391 | 0.814 |
| ACG.R | Anterior cingulate and paracingulate gyri | 0.858 | 1.094 | 0.368 |
| DCG.L | Median cingulate and paracingulate gyri | 1.030 | 1.451 | 0.228 |
| DCG.R | Median cingulate and paracingulate gyri | 1.353 | 1.692 | 0.164 |
| PCG.L | Posterior cingulate gyrus | 0.826 | 0.547 | 0.702 |
| PCG.R | Posterior cingulate gyrus | 1.917 | 1.268 | 0.293 |
| HIP.L | Hippocampus | 0.182 | 0.165 | 0.955 |
| HIP.R | Hippocampus | 0.637 | 0.536 | 0.710 |
| PHG.L | Parahippocampal gyrus | 0.226 | 0.319 | 0.864 |
| PHG.R | Parahippocampal gyrus | 0.709 | 1.059 | 0.385 |
| AMYG.L | Amygdala | 0.678 | 0.799 | 0.531 |
| AMYG.R | Amygdala | 0.016 | 0.067 | 0.992 |
| CAL.L | Calcarine fissure and surrounding cortex | 4.962 | 1.631 | 0.178 |
| CAL.R | Calcarine fissure and surrounding cortex | 1.754 | 0.782 | 0.541 |
| CUN.L | Cuneus | 0.575 | 0.245 | 0.912 |
| CUN.R | Cuneus | 6.345 | 2.789 | **0.034** |
| LING.L | Lingual gyrus | 2.512 | 1.081 | 0.374 |
| LING.R | Lingual gyrus | 2.133 | 1.578 | 0.192 |
| SOG.L | Superior occipital gyrus | 1.948 | 0.661 | 0.622 |
| SOG.R | Superior occipital gyrus | 1.864 | 1.440 | 0.232 |
| MOG.L | Middle occipital gyrus | 1.531 | 1.300 | 0.280 |
| MOG.R | Middle occipital gyrus | 1.940 | 2.337 | 0.065 |
| IOG.L | Inferior occipital gyrus | 0.097 | 0.302 | 0.875 |
| IOG.R | Inferior occipital gyrus | 0.233 | 0.285 | 0.886 |
| FFG.L | Fusiform gyrus | 0.354 | 0.541 | 0.706 |
| FFG.R | Fusiform gyrus | 1.981 | 1.548 | 0.200 |
| PoCG.L | Postcentral gyrus | 0.745 | 0.539 | 0.708 |
| PoCG.R | Postcentral gyrus | 2.737 | 1.349 | 0.262 |
| SPG.L | Superior parietal gyrus | 1.592 | 1.069 | 0.380 |
| SPG.R | Superior parietal gyrus | 4.431 | 1.533 | 0.204 |
| IPL.L | Inferior parietal, but supramarginal and angular gyri | 0.178 | 0.409 | 0.802 |
| IPL.R | Inferior parietal, but supramarginal and angular gyri | 0.514 | 1.073 | 0.378 |
| SMG.L | Supramarginal gyrus | 0.238 | 0.836 | 0.508 |
| SMG.R | Supramarginal gyrus | 0.735 | 1.042 | 0.393 |
| ANG.L | Angular gyrus | 0.066 | 0.135 | 0.969 |
| ANG.R | Angular gyrus | 0.441 | 0.716 | 0.584 |
| PCUN.L | Precuneus | 3.440 | 1.604 | 0.185 |
| PCUN.R | Precuneus | 2.246 | 1.212 | 0.315 |
| PCL.L | Paracentral lobule | 0.484 | 0.646 | 0.632 |
| PCL.R | Paracentral lobule | 0.862 | 1.113 | 0.359 |
| CAU.L | Caudate nucleus | 2.623 | 2.629 | **0.043** |
| CAU.R | Caudate nucleus | 1.115 | 1.207 | 0.317 |
| PUT.L | Lenticular nucleus, putamen | 1.899 | 1.232 | 0.307 |
| PUT.R | Lenticular nucleus, putamen | 9.309 | 3.444 | **0.013** |
| PAL.L | Lenticular nucleus, pallidum | 2.419 | 3.605 | **0.011** |
| PAL.R | Lenticular nucleus, pallidum | 1.808 | 1.767 | 0.147 |
| THA.L | Thalamus | 0.439 | 0.275 | 0.893 |
| THA.R | Thalamus | 1.604 | 1.064 | 0.382 |
| HES.L | Heschl gyrus | 0.177 | 0.922 | 0.457 |
| HES.R | Heschl gyrus | 0.050 | 0.326 | 0.860 |
| STG.L | Superior temporal gyrus | 1.321 | 1.306 | 0.278 |
| STG.R | Superior temporal gyrus | 0.111 | 0.121 | 0.975 |
| TPOsup.L | Temporal pole: superior temporal gyrus | 0.516 | 0.636 | 0.639 |
| TPOsup.R | Temporal pole: superior temporal gyrus | 1.575 | 2.040 | 0.100 |
| MTG.L | Middle temporal gyrus | 5.283 | 3.663 | **0.010** |
| MTG.R | Middle temporal gyrus | 1.851 | 1.258 | 0.296 |
| TPOmid.L | Temporal pole: middle temporal gyrus | 1.421 | 1.071 | 0.379 |
| TPOmid.R | Temporal pole: middle temporal gyrus | 0.792 | 0.782 | 0.542 |
| ITG.L | Inferior temporal gyrus | 2.727 | 1.594 | 0.188 |
| ITG.R | Inferior temporal gyrus | 0.698 | 0.295 | 0.880 |
