# Supplementary material for: White Matter Networks of Phonological Awareness in Chinese Readers
Source: Brain Behav. 2025 Sep 21;15(9):e70781. doi: 10.1002/brb3.70781 (PMC12451024; doi:10.1002/brb3.70781)
Supplement: Supplementary file 2 — Supporting Table S2: brb370781‐sup‐0002‐tableS2.docx [file BRB3-15-e70781-s003.docx]

Table S2: The partial correlation results between nodal degree values and rapid automatized naming (RAN) and character reading phonological awareness (PA).

|  |  | All participants | | | Good readers | | | Poor readers | | |
| --- | --- | --- | --- | --- | --- | --- | --- | --- | --- | --- |
|  |  | RAN | Character reading | PA | RAN | Character reading | PA | RAN | Character reading | PA |
| PreCG.L | r | -0.161 | 0.088 | -0.013 | -0.229 | 0.073 | -0.147 | -0.086 | -0.149 | 0.038 |
|  | p | 0.214 | 0.5 | 0.919 | 0.192 | 0.683 | 0.408 | 0.682 | 0.477 | 0.859 |
| PreCG.R | r | -0.111 | 0.196 | -0.089 | -0.257 | 0.272 | -0.218 | 0.099 | -0.086 | -0.031 |
|  | p | 0.394 | 0.13 | 0.494 | 0.142 | 0.119 | 0.215 | 0.639 | 0.683 | 0.885 |
| SFGdor.L | r | -0.095 | -0.137 | -0.059 | -0.234 | -0.016 | 0.026 | 0.046 | -0.226 | -0.077 |
|  | p | 0.468 | 0.294 | 0.649 | 0.184 | 0.93 | 0.882 | 0.826 | 0.276 | 0.713 |
| SFGdor.R | r | 0.052 | -0.121 | 0.13 | -0.043 | -0.165 | 0.049 | 0.157 | -0.283 | 0.103 |
|  | p | 0.692 | 0.355 | 0.318 | 0.811 | 0.352 | 0.783 | 0.455 | 0.171 | 0.623 |
| ORBsup.L | r | 0.039 | -0.052 | 0.059 | 0.043 | 0.286 | 0.272 | 0.035 | -0.395 | -0.214 |
|  | p | 0.763 | 0.689 | 0.65 | 0.811 | 0.1 | 0.119 | 0.869 | 0.051 | 0.305 |
| ORBsup.R | r | 0.322 | -0.229 | -0.178 | 0.502 | -0.277 | -0.136 | 0.141 | -0.26 | -0.34 |
|  | p | 0.011 | 0.076 | 0.169 | 0.002 | 0.112 | 0.442 | 0.501 | 0.21 | 0.097 |
| MFG.L | r | -0.146 | 0.014 | -0.029 | -0.317 | -0.076 | 0.056 | 0 | 0.031 | -0.27 |
|  | p | 0.263 | 0.915 | 0.823 | 0.068 | 0.668 | 0.751 | 0.999 | 0.884 | 0.192 |
| MFG.R | r | 0.027 | 0.175 | 0.027 | 0.191 | 0.24 | 0.095 | -0.2 | 0.167 | -0.201 |
|  | p | 0.839 | 0.179 | 0.836 | 0.28 | 0.172 | 0.594 | 0.337 | 0.425 | 0.335 |
| ORBmid.L | r | -0.094 | 0.114 | -0.044 | 0.077 | 0.427 | 0.158 | -0.288 | -0.133 | -0.364 |
|  | p | 0.472 | 0.382 | 0.739 | 0.664 | 0.012 | 0.371 | 0.163 | 0.525 | 0.074 |
| ORBmid.R | r | 0.172 | -0.243 | -0.084 | 0.407 | -0.158 | 0.056 | -0.022 | -0.284 | -0.249 |
|  | p | 0.185 | 0.06 | 0.519 | 0.017 | 0.374 | 0.755 | 0.915 | 0.169 | 0.231 |
| IFGoperc.L | r | -0.027 | -0.217 | 0.065 | -0.036 | -0.096 | -0.021 | -0.031 | -0.523 | -0.001 |
|  | p | 0.837 | 0.093 | 0.62 | 0.841 | 0.59 | 0.905 | 0.885 | 0.007 | 0.997 |
| IFGoperc.R | r | -0.001 | -0.064 | 0.04 | -0.072 | -0.103 | 0.256 | 0.066 | -0.238 | -0.398 |
|  | p | 0.997 | 0.622 | 0.758 | 0.686 | 0.563 | 0.143 | 0.755 | 0.252 | 0.049 |
| IFGtriang.L | r | 0.104 | -0.406 | -0.019 | -0.05 | -0.293 | 0.102 | 0.218 | -0.482 | -0.136 |
|  | p | 0.425 | 0.001 | 0.883 | 0.777 | 0.093 | 0.564 | 0.294 | 0.015 | 0.518 |
| IFGtriang.R | r | 0.27 | -0.04 | 0.08 | 0.469 | -0.2 | 0.043 | 0.016 | 0.036 | -0.042 |
|  | p | 0.035 | 0.758 | 0.542 | 0.005 | 0.256 | 0.809 | 0.941 | 0.864 | 0.843 |
| ORBinf.L | r | 0.151 | 0.083 | 0.109 | 0.081 | 0.309 | 0.019 | 0.216 | -0.279 | 0.104 |
|  | p | 0.246 | 0.524 | 0.402 | 0.65 | 0.076 | 0.917 | 0.3 | 0.177 | 0.622 |
| ORBinf.R | r | -0.001 | 0.03 | 0.043 | 0.122 | -0.112 | 0.162 | -0.167 | 0.073 | -0.24 |
|  | p | 0.992 | 0.821 | 0.744 | 0.492 | 0.53 | 0.36 | 0.426 | 0.729 | 0.249 |
| ROL.L | r | -0.156 | -0.119 | 0.114 | -0.045 | 0.028 | 0.357 | -0.323 | -0.249 | -0.288 |
|  | p | 0.229 | 0.362 | 0.381 | 0.799 | 0.873 | 0.038 | 0.116 | 0.23 | 0.163 |
| ROL.R | r | -0.116 | -0.019 | 0.001 | -0.099 | 0.087 | 0.093 | -0.151 | -0.276 | -0.243 |
|  | p | 0.373 | 0.882 | 0.997 | 0.576 | 0.625 | 0.6 | 0.471 | 0.182 | 0.241 |
| SMA.L | r | -0.062 | -0.04 | -0.154 | -0.13 | 0.02 | -0.081 | 0.015 | -0.227 | -0.369 |
|  | p | 0.635 | 0.761 | 0.237 | 0.463 | 0.909 | 0.651 | 0.943 | 0.275 | 0.07 |
| SMA.R | r | -0.208 | -0.003 | -0.049 | -0.216 | 0.057 | -0.051 | -0.203 | -0.075 | -0.104 |
|  | p | 0.107 | 0.979 | 0.708 | 0.219 | 0.747 | 0.772 | 0.331 | 0.721 | 0.62 |
| OLF.L | r | 0.028 | -0.054 | -0.009 | 0.084 | 0.244 | 0.265 | -0.018 | -0.491 | -0.368 |
|  | p | 0.828 | 0.678 | 0.946 | 0.635 | 0.164 | 0.129 | 0.931 | 0.013 | 0.071 |
| OLF.R | r | 0.276 | 0.089 | -0.171 | 0.261 | 0.38 | -0.072 | 0.296 | -0.196 | -0.264 |
|  | p | 0.032 | 0.494 | 0.187 | 0.137 | 0.027 | 0.686 | 0.15 | 0.348 | 0.203 |
| SFGmed.L | r | 0.129 | -0.089 | 0.198 | 0.136 | -0.191 | 0.171 | 0.147 | -0.217 | 0.095 |
|  | p | 0.323 | 0.498 | 0.125 | 0.443 | 0.279 | 0.333 | 0.483 | 0.298 | 0.652 |
| SFGmed.R | r | 0.09 | -0.024 | 0.103 | 0.191 | -0.122 | 0.201 | -0.029 | 0.041 | -0.093 |
|  | p | 0.491 | 0.857 | 0.43 | 0.279 | 0.493 | 0.254 | 0.89 | 0.844 | 0.657 |
| ORBsupmed.L | r | 0.022 | -0.162 | 0.055 | 0.183 | -0.392 | 0.101 | -0.085 | -0.112 | -0.014 |
|  | p | 0.865 | 0.213 | 0.676 | 0.299 | 0.022 | 0.57 | 0.685 | 0.595 | 0.948 |
| ORBsupmed.R | r | 0.164 | -0.166 | -0.057 | 0.208 | -0.155 | 0.226 | 0.131 | -0.076 | -0.226 |
|  | p | 0.207 | 0.2 | 0.662 | 0.238 | 0.382 | 0.199 | 0.531 | 0.719 | 0.277 |
| REC.L | r | 0.027 | -0.092 | -0.15 | 0.013 | 0.112 | 0.078 | 0.038 | -0.269 | -0.404 |
|  | p | 0.835 | 0.48 | 0.248 | 0.943 | 0.527 | 0.661 | 0.858 | 0.194 | 0.045 |
| REC.R | r | 0.084 | -0.076 | 0.052 | 0.029 | -0.171 | 0.157 | 0.144 | -0.108 | -0.146 |
|  | p | 0.518 | 0.562 | 0.691 | 0.873 | 0.334 | 0.374 | 0.492 | 0.606 | 0.486 |
| INS.L | r | 0.023 | -0.069 | 0.006 | 0.14 | 0.127 | 0.18 | -0.104 | -0.359 | -0.261 |
|  | p | 0.858 | 0.598 | 0.962 | 0.431 | 0.473 | 0.307 | 0.621 | 0.078 | 0.207 |
| INS.R | r | 0.18 | -0.01 | 0.049 | 0.432 | 0.011 | 0.023 | -0.058 | -0.284 | 0.058 |
|  | p | 0.166 | 0.941 | 0.706 | 0.011 | 0.951 | 0.896 | 0.782 | 0.169 | 0.784 |
| ACG.L | r | 0.103 | 0.088 | 0.029 | 0.383 | -0.057 | 0.2 | -0.176 | 0.01 | -0.333 |
|  | p | 0.428 | 0.499 | 0.824 | 0.025 | 0.748 | 0.257 | 0.4 | 0.961 | 0.104 |
| ACG.R | r | 0.175 | 0.19 | 0.057 | 0.158 | 0.242 | 0.392 | 0.213 | 0.063 | -0.442 |
|  | p | 0.177 | 0.143 | 0.661 | 0.371 | 0.169 | 0.022 | 0.307 | 0.765 | 0.027 |
| DCG.L | r | 0.154 | 0.01 | 0.218 | 0.103 | -0.09 | 0.279 | 0.251 | -0.256 | -0.117 |
|  | p | 0.237 | 0.937 | 0.091 | 0.562 | 0.613 | 0.111 | 0.226 | 0.217 | 0.578 |
| DCG.R | r | 0.006 | 0.099 | 0.087 | -0.089 | 0.154 | 0.254 | 0.09 | -0.203 | -0.175 |
|  | p | 0.961 | 0.449 | 0.505 | 0.617 | 0.385 | 0.147 | 0.67 | 0.331 | 0.402 |
| PCG.L | r | 0.226 | -0.122 | 0.094 | 0.15 | -0.094 | 0.198 | 0.338 | -0.43 | -0.176 |
|  | p | 0.08 | 0.349 | 0.471 | 0.397 | 0.597 | 0.26 | 0.098 | 0.032 | 0.399 |
| PCG.R | r | 0.185 | 0.008 | 0.128 | 0.118 | 0.273 | 0.317 | 0.267 | -0.272 | -0.057 |
|  | p | 0.153 | 0.952 | 0.326 | 0.505 | 0.118 | 0.068 | 0.196 | 0.188 | 0.788 |
| HIP.L | r | 0.141 | 0.08 | 0.106 | 0.208 | 0.151 | 0.243 | 0.086 | 0.026 | 0.033 |
|  | p | 0.28 | 0.54 | 0.416 | 0.237 | 0.395 | 0.165 | 0.683 | 0.903 | 0.877 |
| HIP.R | r | 0.168 | 0.031 | 0.147 | 0.091 | -0.063 | 0.294 | 0.262 | -0.075 | -0.11 |
|  | p | 0.197 | 0.812 | 0.258 | 0.608 | 0.724 | 0.091 | 0.207 | 0.721 | 0.6 |
| PHG.L | r | 0.103 | -0.031 | 0.17 | 0.283 | 0.013 | 0.173 | -0.121 | -0.173 | 0.312 |
|  | p | 0.43 | 0.812 | 0.191 | 0.105 | 0.943 | 0.328 | 0.563 | 0.409 | 0.129 |
| PHG.R | r | -0.001 | 0.009 | 0.102 | -0.055 | -0.007 | 0.13 | 0.056 | -0.019 | 0.155 |
|  | p | 0.992 | 0.946 | 0.432 | 0.759 | 0.967 | 0.464 | 0.789 | 0.929 | 0.461 |
| AMYG.L | r | -0.071 | 0.296 | 0.366 | 0.016 | 0.325 | 0.335 | -0.17 | 0.28 | 0.456 |
|  | p | 0.584 | 0.021 | 0.004 | 0.93 | 0.061 | 0.053 | 0.415 | 0.175 | 0.022 |
| AMYG.R | r | -0.085 | 0.103 | 0.348 | -0.074 | 0.254 | 0.395 | -0.104 | 0.029 | 0.44 |
|  | p | 0.514 | 0.431 | 0.006 | 0.676 | 0.148 | 0.021 | 0.621 | 0.889 | 0.028 |
| CAL.L | r | -0.048 | 0.066 | 0.153 | -0.132 | 0.193 | 0.076 | 0.05 | -0.014 | 0.261 |
|  | p | 0.715 | 0.611 | 0.238 | 0.456 | 0.274 | 0.67 | 0.813 | 0.947 | 0.208 |
| CAL.R | r | 0.34 | 0.13 | 0.028 | 0.436 | -0.076 | -0.023 | 0.281 | 0.181 | -0.126 |
|  | p | 0.007 | 0.316 | 0.832 | 0.01 | 0.668 | 0.897 | 0.173 | 0.388 | 0.547 |
| CUN.L | r | 0.227 | 0.211 | 0.133 | 0.111 | 0.273 | 0.094 | 0.423 | -0.027 | 0.054 |
|  | p | 0.078 | 0.103 | 0.305 | 0.532 | 0.118 | 0.598 | 0.035 | 0.896 | 0.799 |
| CUN.R | r | -0.019 | 0.21 | 0.08 | 0.052 | -0.052 | -0.174 | -0.089 | 0.262 | 0.269 |
|  | p | 0.887 | 0.105 | 0.538 | 0.769 | 0.771 | 0.326 | 0.672 | 0.206 | 0.193 |
| LING.L | r | 0.269 | 0.204 | 0.15 | 0.387 | 0.2 | 0.199 | 0.115 | 0.308 | 0.014 |
|  | p | 0.036 | 0.115 | 0.248 | 0.024 | 0.256 | 0.259 | 0.584 | 0.135 | 0.948 |
| LING.R | r | 0.122 | 0.103 | 0.072 | 0.264 | 0.043 | 0.116 | -0.038 | 0.323 | 0.091 |
|  | p | 0.351 | 0.431 | 0.583 | 0.132 | 0.81 | 0.512 | 0.856 | 0.115 | 0.667 |
| SOG.L | r | -0.104 | 0.138 | 0.233 | 0.127 | 0.073 | 0.172 | -0.31 | -0.023 | 0.19 |
|  | p | 0.423 | 0.29 | 0.071 | 0.475 | 0.68 | 0.33 | 0.131 | 0.915 | 0.362 |
| SOG.R | r | 0.133 | 0.136 | 0.147 | 0.096 | 0.108 | 0.199 | 0.22 | -0.056 | -0.164 |
|  | p | 0.307 | 0.295 | 0.259 | 0.588 | 0.543 | 0.258 | 0.291 | 0.79 | 0.433 |
| MOG.L | r | 0.052 | 0 | 0.19 | 0.118 | 0.164 | 0.305 | -0.026 | -0.385 | -0.07 |
|  | p | 0.691 | 1 | 0.142 | 0.507 | 0.355 | 0.079 | 0.901 | 0.057 | 0.739 |
| MOG.R | r | -0.171 | 0.076 | 0.33 | -0.158 | 0.068 | 0.35 | -0.211 | -0.071 | 0.151 |
|  | p | 0.187 | 0.563 | 0.009 | 0.371 | 0.703 | 0.043 | 0.311 | 0.738 | 0.472 |
| IOG.L | r | 0.16 | 0.033 | 0.019 | -0.114 | 0.096 | 0.022 | 0.437 | -0.211 | -0.102 |
|  | p | 0.219 | 0.799 | 0.883 | 0.521 | 0.588 | 0.902 | 0.029 | 0.31 | 0.629 |
| IOG.R | r | -0.044 | 0.139 | -0.107 | -0.022 | 0.055 | -0.302 | -0.073 | 0.112 | 0.061 |
|  | p | 0.738 | 0.284 | 0.412 | 0.9 | 0.757 | 0.083 | 0.728 | 0.593 | 0.771 |
| FFG.L | r | -0.082 | 0.288 | 0.449 | -0.059 | 0.336 | 0.456 | -0.147 | 0.202 | 0.456 |
|  | p | 0.53 | 0.025 | **0.000287** | 0.741 | 0.052 | 0.007 | 0.484 | 0.333 | 0.022 |
| FFG.R | r | 0.039 | -0.084 | 0.116 | 0.137 | -0.15 | 0.021 | -0.078 | -0.016 | 0.247 |
|  | p | 0.767 | 0.522 | 0.372 | 0.44 | 0.398 | 0.905 | 0.712 | 0.941 | 0.234 |
| PoCG.L | r | 0.138 | 0.006 | -0.106 | 0.26 | -0.041 | 0.145 | 0.057 | -0.069 | -0.356 |
|  | p | 0.289 | 0.961 | 0.414 | 0.137 | 0.818 | 0.415 | 0.788 | 0.745 | 0.081 |
| PoCG.R | r | -0.114 | 0.048 | -0.137 | -0.121 | 0.137 | 0.208 | -0.107 | -0.084 | -0.444 |
|  | p | 0.384 | 0.711 | 0.291 | 0.496 | 0.44 | 0.238 | 0.611 | 0.69 | 0.026 |
| SPG.L | r | 0.275 | -0.042 | 0.125 | -0.012 | -0.173 | 0.158 | 0.562 | 0.038 | 0.059 |
|  | p | 0.032 | 0.75 | 0.336 | 0.944 | 0.327 | 0.374 | 0.003 | 0.857 | 0.778 |
| SPG.R | r | -0.207 | 0.098 | 0.298 | -0.197 | -0.09 | 0.481 | -0.229 | 0.257 | -0.017 |
|  | p | 0.11 | 0.452 | 0.019 | 0.265 | 0.615 | 0.004 | 0.272 | 0.215 | 0.934 |
| IPL.L | r | -0.165 | -0.086 | 0.043 | -0.072 | 0.014 | 0.142 | -0.32 | -0.231 | -0.111 |
|  | p | 0.203 | 0.512 | 0.745 | 0.684 | 0.935 | 0.424 | 0.119 | 0.266 | 0.598 |
| IPL.R | r | -0.031 | 0.14 | -0.039 | -0.116 | 0.097 | -0.045 | 0.075 | -0.136 | -0.389 |
|  | p | 0.812 | 0.281 | 0.763 | 0.515 | 0.587 | 0.8 | 0.721 | 0.517 | 0.055 |
| SMG.L | r | -0.034 | -0.028 | -0.05 | 0.178 | 0.235 | 0.042 | -0.231 | -0.294 | -0.207 |
|  | p | 0.795 | 0.831 | 0.702 | 0.314 | 0.181 | 0.815 | 0.267 | 0.154 | 0.321 |
| SMG.R | r | 0.079 | 0.017 | 0.206 | 0.105 | -0.005 | 0.102 | 0.058 | -0.085 | 0.272 |
|  | p | 0.546 | 0.895 | 0.112 | 0.555 | 0.976 | 0.566 | 0.785 | 0.685 | 0.188 |
| ANG.L | r | -0.171 | 0.063 | 0.137 | -0.282 | -0.02 | 0.112 | -0.037 | -0.038 | 0.088 |
|  | p | 0.188 | 0.627 | 0.292 | 0.106 | 0.91 | 0.527 | 0.861 | 0.857 | 0.675 |
| ANG.R | r | -0.081 | 0.022 | 0.053 | -0.076 | 0.098 | 0.126 | -0.083 | -0.184 | -0.081 |
|  | p | 0.533 | 0.867 | 0.688 | 0.671 | 0.583 | 0.479 | 0.693 | 0.379 | 0.7 |
| PCUN.L | r | -0.039 | -0.018 | 0.162 | -0.116 | -0.17 | 0.101 | 0.061 | 0.018 | 0.128 |
|  | p | 0.767 | 0.89 | 0.212 | 0.515 | 0.337 | 0.572 | 0.772 | 0.933 | 0.542 |
| PCUN.R | r | 0.163 | 0.019 | 0.088 | 0.212 | 0.107 | 0.111 | 0.118 | -0.223 | -0.077 |
|  | p | 0.208 | 0.882 | 0.502 | 0.229 | 0.547 | 0.532 | 0.574 | 0.284 | 0.716 |
| PCL.L | r | -0.19 | -0.131 | -0.047 | -0.358 | -0.067 | 0.122 | -0.042 | -0.339 | -0.235 |
|  | p | 0.142 | 0.316 | 0.72 | 0.038 | 0.704 | 0.492 | 0.842 | 0.097 | 0.259 |
| PCL.R | r | 0.214 | 0.082 | 0.185 | 0.141 | 0.244 | -0.017 | 0.301 | -0.137 | 0.267 |
|  | p | 0.097 | 0.531 | 0.153 | 0.426 | 0.163 | 0.924 | 0.143 | 0.512 | 0.197 |
| CAU.L | r | 0.214 | -0.071 | -0.025 | 0.202 | -0.137 | 0.134 | 0.247 | 0.125 | -0.107 |
|  | p | 0.098 | 0.586 | 0.85 | 0.252 | 0.441 | 0.45 | 0.234 | 0.551 | 0.609 |
| CAU.R | r | 0.071 | -0.134 | -0.147 | -0.025 | 0.121 | -0.072 | 0.207 | -0.333 | -0.13 |
|  | p | 0.587 | 0.305 | 0.259 | 0.888 | 0.494 | 0.685 | 0.322 | 0.103 | 0.536 |
| PUT.L | r | -0.028 | 0.294 | 0.229 | -0.033 | 0.432 | 0.362 | -0.021 | 0.13 | -0.001 |
|  | p | 0.831 | 0.022 | 0.076 | 0.853 | 0.011 | 0.035 | 0.922 | 0.534 | 0.997 |
| PUT.R | r | -0.145 | 0.109 | 0.3 | -0.208 | 0.094 | 0.291 | -0.099 | 0.066 | 0.285 |
|  | p | 0.266 | 0.401 | 0.019 | 0.238 | 0.596 | 0.095 | 0.639 | 0.754 | 0.168 |
| PAL.L | r | 0.003 | -0.111 | -0.051 | -0.146 | -0.13 | 0.05 | 0.228 | -0.235 | -0.353 |
|  | p | 0.982 | 0.394 | 0.697 | 0.41 | 0.465 | 0.777 | 0.273 | 0.258 | 0.083 |
| PAL.R | r | -0.022 | 0.18 | 0.168 | -0.065 | 0.143 | 0.113 | 0.038 | 0.31 | 0.389 |
|  | p | 0.868 | 0.164 | 0.196 | 0.715 | 0.42 | 0.525 | 0.857 | 0.132 | 0.055 |
| THA.L | r | 0.179 | 0.054 | 0.156 | 0.157 | -0.238 | -0.045 | 0.214 | 0.344 | 0.41 |
|  | p | 0.169 | 0.68 | 0.231 | 0.374 | 0.175 | 0.802 | 0.305 | 0.092 | 0.042 |
| THA.R | r | 0.044 | 0.11 | 0.027 | 0.216 | -0.055 | -0.081 | -0.121 | 0.246 | 0.158 |
|  | p | 0.737 | 0.4 | 0.834 | 0.22 | 0.757 | 0.648 | 0.565 | 0.235 | 0.451 |
| HES.L | r | -0.094 | -0.006 | -0.062 | -0.147 | 0.042 | -0.009 | -0.043 | 0.044 | -0.007 |
|  | p | 0.47 | 0.961 | 0.634 | 0.406 | 0.812 | 0.961 | 0.839 | 0.834 | 0.972 |
| HES.R | r | 0.096 | 0.096 | 0.1 | 0.112 | 0.334 | 0.173 | 0.081 | 0.062 | 0.216 |
|  | p | 0.463 | 0.46 | 0.444 | 0.529 | 0.054 | 0.327 | 0.701 | 0.768 | 0.3 |
| STG.L | r | -0.039 | -0.092 | 0.22 | -0.008 | -0.07 | 0.269 | -0.081 | -0.106 | 0.157 |
|  | p | 0.763 | 0.482 | 0.089 | 0.963 | 0.694 | 0.124 | 0.699 | 0.616 | 0.453 |
| STG.R | r | 0.052 | -0.002 | 0.035 | 0.001 | -0.173 | 0.208 | 0.121 | 0.198 | -0.241 |
|  | p | 0.692 | 0.988 | 0.788 | 0.997 | 0.327 | 0.238 | 0.565 | 0.342 | 0.245 |
| TPOsup.L | r | -0.124 | 0.153 | 0.396 | -0.02 | 0.072 | 0.283 | -0.248 | 0.233 | 0.506 |
|  | p | 0.341 | 0.24 | 0.002 | 0.912 | 0.685 | 0.105 | 0.232 | 0.261 | 0.01 |
| TPOsup.R | r | -0.078 | -0.019 | 0.066 | -0.064 | 0.089 | 0.13 | -0.098 | -0.151 | -0.062 |
|  | p | 0.55 | 0.882 | 0.613 | 0.721 | 0.615 | 0.464 | 0.64 | 0.472 | 0.768 |
| MTG.L | r | 0.016 | 0.056 | 0.56 | -0.055 | -0.001 | 0.564 | 0.109 | 0.088 | 0.545 |
|  | p | 0.905 | 0.667 | **0.000003** | 0.759 | 0.995 | 0.001 | 0.603 | 0.675 | 0.005 |
| MTG.R | r | 0.094 | 0.055 | -0.1 | 0.367 | -0.134 | -0.111 | -0.14 | 0.236 | -0.098 |
|  | p | 0.47 | 0.674 | 0.442 | 0.033 | 0.451 | 0.532 | 0.504 | 0.257 | 0.64 |
| TPOmid.L | r | 0.032 | 0.3 | 0.346 | 0.261 | 0.286 | 0.333 | -0.226 | 0.269 | 0.352 |
|  | p | 0.805 | 0.019 | 0.006 | 0.135 | 0.101 | 0.054 | 0.277 | 0.194 | 0.084 |
| TPOmid.R | r | -0.083 | 0.203 | 0.136 | -0.136 | 0.349 | 0.251 | -0.037 | -0.013 | 0.026 |
|  | p | 0.523 | 0.116 | 0.296 | 0.444 | 0.043 | 0.153 | 0.86 | 0.952 | 0.901 |
| ITG.L | r | 0.022 | 0.114 | 0.082 | 0.063 | 0.293 | 0.179 | -0.019 | -0.173 | -0.097 |
|  | p | 0.864 | 0.382 | 0.528 | 0.722 | 0.093 | 0.312 | 0.928 | 0.408 | 0.646 |
| ITG.R | r | 0.028 | 0.061 | 0.031 | 0.244 | -0.077 | -0.103 | -0.144 | 0.078 | 0.069 |
|  | p | 0.832 | 0.638 | 0.815 | 0.164 | 0.663 | 0.562 | 0.493 | 0.712 | 0.744 |
