# Supplementary material for: White Matter Networks of Phonological Awareness in Chinese Readers
Source: Brain Behav. 2025 Sep 21;15(9):e70781. doi: 10.1002/brb3.70781 (PMC12451024; doi:10.1002/brb3.70781)
Supplement: Supplementary file 3 — Supplementary information: brb370781‐sup‐0003‐SuppMat.docx [file BRB3-15-e70781-s001.docx]

**Nodal Degrees Analysis**

**1 Methods**

The remaining methods were the same as our manuscript. We defined the backbone network based on the all participants. For each potential pair of cortical nodes, we applied a one-tailed nonparametric sign test under the null hypothesis of no connection between them (i.e., fibre bundle count = zero). Bonferroni correction was applied for multiple comparisons (C_90_^2^ = 4005 pairs of regions, *p* < 0.05/4005 ≈ 1.25×10⁻^5^) to create a symmetric binarized matrix that preserved 2082 tracts. The finished network has 25.7% sparsity, which is within a range shown to support stable graph metrics in structural brain networks [(Dennis et al., 2012)](https://link.springer.com/chapter/10.1007/978-3-642-33454-2_38). The mean FA of the backbone network was extracted for both the poor readers and good readers groups using the backbone network mask derived from the all participants. The statistical analyses were the same as our manuscript.

**2 Results**

2.1 Group differences in white matter

ANCOVA was performed on the nodal degree values of 90 AAL areas in the weighted white matter network of poor and good readers to compare brain white matter structures between groups. After controlling for sex, age, and non-verbal IQ, none of these regions’ nodal degree comparisons across groups survived after BH FDR correction (q < 0.05).

2.2 Partial correlation between nodal degree values and reading abilities

Since there was no group difference between the two groups, partial correlations were computed for all participants between character reading, PA, and RAN z-scores and nodal degree values of the 90 AAL regions. Nodal degree values of one AAL region was significantly positively linked with PA after BH FDR correction (controlling for sex, age, and non-verbal IQ): left MTG (*r* = 0.572, *p* = 0.000001, FDR-adjusted *p* = 0.00009). None of these regions’ nodal degree was linked with character reading and RAN.

2.3 Regression analysis

We conducted a hierarchical linear regression analysis to further evaluate the relationship between the left MTG and PA. PA of the entire sample was the regression model's dependent variable. Control factors included gender, age, and non-verbal IQ. Left MTG nodal degree values were entered into the model concurrently as independent variables in the second stage. After controlling for sex, age, and non-verbal IQ (*ΔR²* = .100), significant associations were observed between PA and left MTG nodal degrees (*ΔR²* = .294; *β* = .556, *p* <.001).

2.4 Mediation analysis

A mediation model was tested, in which nodal degrees of left MTG were independent variables. In the mediation model, PA was mediator and character reading was dependent variable. Age, sex, and IQ were covariates. Our results found that the mediation effect of PA between the left MTG on character reading was significant (*β* = .287 , 95% CI [.103, .474]). In addition, the direct effect from the left MTG to character reading was not significant in the mediation model (*β* = -.065, 95% CI [-.244, .152]), suggesting full mediation effect of PA. In other words, the association between the left MTG and character reading appeared to be accounted for by PA.

**Network-Based Statistic**

To examine group differences in white matter networks, we performed a Network-Based Statistic (NBS) analysis (Zalesky et al., 2010). The input for the analysis was a 90 × 90 fiber numbers (FN) structural connectivity matrix for each participant, generated by using PANDA toolbox (Cui et al., 2013). Each matrix element represented the number of streamlines connecting pairs of AAL-defined brain regions, as determined by deterministic fiber tracking.

Firstly, we conducted a general linear model (GLM) was constructed, with different group as the main variable and age, sex, and IQ included as covariates. Group differences in each connection were examined based on a two-tailed test and the contrast vector [1, 0, 0, 0, 0], to test for group differences in connection strength without assuming a direction.

Next, a primary threshold (t > 2.5) was applied to the t-statistic matrix to identify suprathreshold links. We used breadth-first search (Ahuja et al., 1993) to detect connected components among these links, each representing a candidate subnetwork, with its size defined by the number of edges it contained. To ascribe a p value controlled for the family-wise error (FWE) rate to each component, permutation testing was conducted based on each component's size. Five thousand permutations of group membership were generated independently. the size of the largest subnetwork in each permutation was recorded to create a null distribution of maximal component size. The observed subnetworks were then compared against this null distribution to calculate family-wise error (FWE) corrected p-values. Subnetworks with FWE-corrected p < 0.05 were considered statistically significant.

The NBS analysis did not reveal any significant group differences in white matter connectivity. With an initial threshold of t > 2.5 and 5,000 permutations, no subnetworks survived FWE correction at p < 0.05. This result was consistent with the analysis of nodal degrees, which also showed no significant group effects.

References:

Ahuja, R. K., Magnanti, T. L., & Orlin, J. B. (1993). Network flows: Theory, algorithms, and applications. Journal of the Operational Research Society, 45, 791–796. 10.1016/0166-218X(94)90171-6.

Cui, Z., Zhong, S., Xu, P., He, Y., & Gong, G. (2013). PANDA: A pipeline toolbox for analyzing brain diffusion images. Frontiers in Human Neuroscience, 7, 42. <https://doi.org/10.3389/fnhum.2013.00042>

Dennis, E. L., Jahanshad, N., Toga, A. W., McMahon, K. L., de Zubicaray, G. I., Martin, N. G., Wright, M. J., & Thompson, P. M. (2012). Test-retest reliability of graph theory measures of structural brain connectivity. Medical image computing and computer-assisted intervention : MICCAI ... International Conference on Medical Image Computing and Computer-Assisted Intervention, 15(Pt 3), 305–312. https://doi.org/10.1007/978-3-642-33454-2_38

Liu, T., Thiebaut de Schotten, M., Altarelli, I., Ramus, F., & Zhao, J. (2021). Maladaptive compensation of right fusiform gyrus in developmental dyslexia: A hub-based white matter network analysis. Cortex, 145, 57-66. https://doi.org/10.1016/j.cortex.2021.07.016

Liu, T., Thiebaut de Schotten, M., Altarelli, I., Ramus, F., & Zhao, J. (2022). Neural dissociation of visual attention span and phonological deficits in developmental dyslexia: A hub-based white matter network analysis. Human Brain Mapping, 43(17), 5210-5219. https://doi.org/10.1002/hbm.25997

Zalesky, A., Fornito, A., & Bullmore, E. T. (2010). Network-based statistic: identifying differences in brain networks. NeuroImage, 53(4), 1197–1207. https://doi.org/10.1016/j.neuroimage.2010.06.041
